# In-Depth Serum Proteomics Reveals the Trajectory of Hallmarks of Cancer in Hepatitis B Virus–Related Liver Diseases

**DOI:** 10.1016/j.mcpro.2023.100574

**Published:** 2023-05-19

**Authors:** Meng Xu, Kaikun Xu, Shangqi Yin, Cheng Chang, Wei Sun, Guibin Wang, Kai Zhang, Jinsong Mu, Miantao Wu, Baocai Xing, Xiaomei Zhang, Jinyu Han, Xiaohang Zhao, Yajie Wang, Danke Xu, Xiaobo Yu

**Affiliations:** 1 State Key Laboratory of Analytical Chemistry for Life Science, School of Chemistry and Chemical Engineering, Nanjing University, Nanjing, China; 2 State Key Laboratory of Proteomics, Beijing Proteome Research Center, National Center for Protein Sciences, Beijing Institute of Lifeomics, Beijing, China; 3 Research Unit of Proteomics Driven Cancer Precision Medicine, Chinese Academy of Medical Sciences, Beijing, China; 4 Department of Clinical Laboratory, Beijing Ditan Hospital, Capital Medical University, Beijing, China; 5 Department of Critical Care Medicine, The Fifth Medical Center, Chinese PLA General Hospital, Beijing, China; 6 Sun Yat-sen University Cancer Center, State Key Laboratory of Oncology in South China, Collaborative Innovation Center for Cancer Medicine, Guangzhou, China; 7 Department of Hepato-Pancreato-Biliary Surgery I, Key Laboratory of Carcinogenesis and Translational Research (Ministry of Education/Beijing), Peking University Cancer Hospital and Institute, Beijing, China; 8 State Key Laboratory of Molecular Oncology, Cancer Hospital, Chinese Academy of Medical Sciences and Peking Union Medical College, Beijing, China

**Keywords:** hepatocellular carcinoma, mass spectrometry, antibody array, biomarker, drug target

## Abstract

Hepatocellular carcinoma (HCC) is a prevalent cancer in China, with chronic hepatitis B (CHB) and liver cirrhosis (LC) being high-risk factors for developing HCC. Here, we determined the serum proteomes (762 proteins) of 125 healthy controls and Hepatitis B virus–infected CHB, LC, and HCC patients and constructed the first cancerous trajectory of liver diseases. The results not only reveal that the majority of altered biological processes were involved in the hallmarks of cancer (inflammation, metastasis, metabolism, vasculature, and coagulation) but also identify potential therapeutic targets in cancerous pathways (*i.e.*, IL17 signaling pathway). Notably, the biomarker panels for detecting HCC in CHB and LC high-risk populations were further developed using machine learning in two cohorts comprised of 200 samples (discovery cohort = 125 and validation cohort = 75). The protein signatures significantly improved the area under the receiver operating characteristic curve of HCC (CHB discovery and validation cohort = 0.953 and 0.891, respectively; LC discovery and validation cohort = 0.966 and 0.818, respectively) compared to using the traditional biomarker, alpha-fetoprotein, alone. Finally, selected biomarkers were validated with parallel reaction monitoring mass spectrometry in an additional cohort (n = 120). Altogether, our results provide fundamental insights into the continuous changes of cancer biology processes in liver diseases and identify candidate protein targets for early detection and intervention.

## Highlights

• Determined the serum proteomes of liver diseases using DIA-MS and antibody arrays.
• Constructed the first cancerous trajectory of liver diseases.
• Identified biomarker panels for LC and HCC patients that are superior to AFP.

## Graphical Abstract

**Figure:**
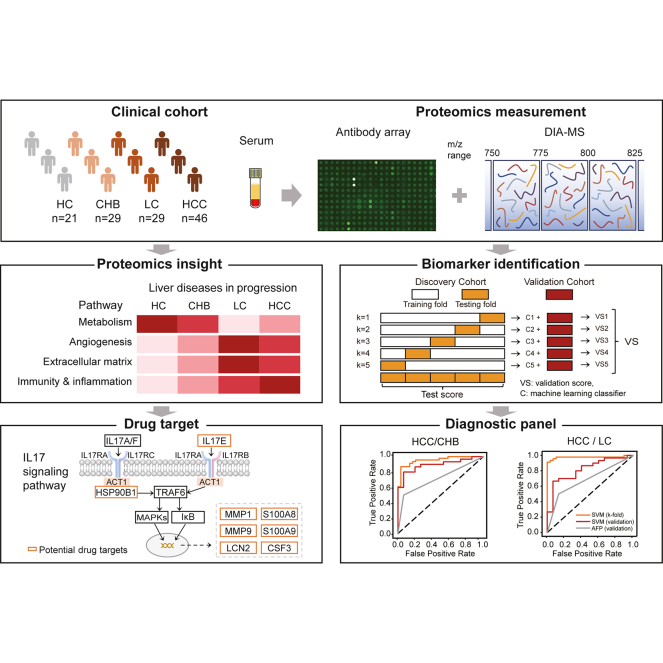

## In Brief

We determined the serum proteomes of 125 healthy controls and Hepatitis B virus-infected CHB, LC, and HCC patients using DIA-MS and customized antibody microarrays, and built the first cancerous trajectory of liver diseases. The results revealed the altered biological processes involved in the hallmarks of cancer and identified potential therapeutic targets in cancerous pathways. Notably, the biomarker panels for detecting HCC in CHB and LC high-risk populations were further developed using machine learning with significantly improved performance compared to AFP.

Tumorigenesis is a continuum in which cells transition from normal to dysregulated to cancerous. A critical challenge is to understand the biology of this trajectory, which is valuable in early detection and intervention (1). For example, hepatocellular carcinoma (HCC) is one of the most prevalent types of cancer worldwide and ranks fourth globally for cancer fatality rate (2, 3). In China, 85% of HCC cases are caused by hepatitis B virus (HBV) infection (4). Chronic hepatitis B (CHB) and HBV-related liver cirrhosis (LC) are the leading risk factors of HCC and represent increasing severity and progression of liver diseases (5, 6, 7). In a prospective cohort study, 105 untreated CHB patients without LC at diagnosis were followed for ∼23 years. The hazard ratio for LC occurrence was 7-fold higher in patients of active hepatitis than inactive carriers, and the LC occurrence significantly increased the risk of HCC (hazard ratio 20.4, 95% confidence interval 2.54–167.5) and liver-related death (hazard ratio 16.5, 95% confidence interval 2.0–138.8) (8). However, the molecular mechanisms that drive the progression of liver disease from normal to CHB to LC and, finally, to HCC are unclear due to the lack of functional studies with appropriate clinical samples.

Alpha-fetoprotein (AFP) is the standard biomarker for HCC diagnostics, with a reported sensitivity ranging from 40% to 60% and a specificity ranging from 80% to 90% (9). Although new candidate biomarkers (*e.g.*, AFP-L3, DCP) may improve HCC detection when used in conjunction with AFP, the sensitivity and specificity remain unsatisfactory (10, 11, 12).

Blood contains circulating proteins that are crucial in modulating biological functions, such as inflammation, immunity, coagulation, and metabolism. Therefore, measuring the protein expression changes in serological proteomes during disease progression can provide valuable insight into the mechanisms of human physiology and pathology (13, 14). Using high-abundant protein depletion, isoelectric focusing-SDS-PAGE, and liquid chromatography/electrospray ionization quadrupole time-of-flight mass spectrometry (MS), Fye *et al.* analyzed the differential expression of proteins in pooled plasma samples taken from 339 healthy controls (HC), LC, and HCC patients. Twenty-six differentially expressed proteins (DEPs) were identified among three groups, of which four potential biomarkers (hemopexin, alpha-1-antitrypsin, apolipoprotein A1, and complement component 3) were validated using ELISA (15). Using high-abundant protein depletion and liquid chromatography-MS (LC-MS/MS), Tsai *et al.* analyzed proteins in the serum of 205 LC and HCC patients from two independent cohorts and detected 269 and 252 proteins, respectively. Twenty-one potential biomarkers that were enriched in the complement and coagulation cascades and antigen processing and presentation pathways were validated using multiple reaction monitoring-MS (16). Using targeted multiple reaction monitoring-MS, Yeo *et al.* identified a 28-protein signature of CHB, LC, and HCC patients, which was developed and validated in training (n = 713) and validation (n = 305) sample sets. Compared to AFP, the area under the receiver operating characteristic curve (AUC) of this multimarker panel significantly increased in the training (0.976 *versus* 0.804; *p* < 0.001) and validation (0.898 *versus* 0.778; *p* < 0.001) sets (17). These results indicate that serum proteomics has great potential in detecting liver diseases. However, these studies depleted highly abundant proteins in serum, which could disrupt the proteome *via* the concomitant loss of lower abundance proteins during the depletion process. In addition, the appropriate control group (*i.e.*, CHB for Fye’s study, HC and HCB for Tai’s study, and HC group for Yeo’s study) was not employed. Therefore, systematic analyses of liver disease from HC to HCC were not performed properly.

Compared to prior studies, we analyzed the proteomes of 125 serum samples from patients representing the liver disease progression (HC, CHB, LC, and HCC) using our in-depth serum proteome mapping (ID-Map) platform that combines high-density antibody microarray with data-independent acquisition mass spectrometry (DIA-MS) (18). This approach can detect over 700 nonredundant, low abundance proteins with concentrations spanning 10∼12 orders in magnitude without depleting the high abundance proteins (19, 20, 21, 22). In addition, the proteome changes revealed altered biology processes and signaling pathways that occur during the HC-CHB-LC-HCC progression. Finally, biomarker signatures specific to CHB, LC, and HCC were identified by machine learning in discovery and validation cohorts. The protein signatures resulted in superior sensitivity and specificity compared to AFP alone.

## Experimental Procedures

### Clinical Cohort

Three clinical cohorts were collected in this study. Sera from 21 HCs in the discovery cohort were obtained from Cancer Hospital, Chinese Academy of Medical Sciences and Peking Union Medical College. Sera from 29 CHB and 29 LC patients were collected from the Fifth Medical Center, Chinese PLA General Hospital, and 46 HCC patients before surgery were obtained from Sun Yat-sen University Cancer Center or Peking University Cancer Hospital (Table 1). The validation cohort was comprised of 75 serum samples collected from Beijing Ditan Hospital, Capital Medical University, including 15 cases of HCs, 15 CHB patients, 15 LC patients, and 30 HCC patients (Table 2). Another additional cohort comprised of 120 serum samples were collected from Beijing Ditan Hospital, Capital Medical University, including 20 cases of HCs, 25 CHB patients, 30 LC patients, and 45 HCC patients (Table 3). All liver disease (CHB, LC, and HCC) patients were hepatitis B surface antigen-positive and/or hepatitis B core antibody-positive with an HBV infection history. All samples were stored at −80 °C. This research was approved by the Ethics Committee of Peking University Cancer Hospital and Beijing Ditan Hospital (No. 2019-039-03), and an exemption of informed consent was obtained prior to sera collection. All experiments were performed according to the standards of the Declaration of Helsinki.

**Table 1:** Patient baseline characteristics of the discovery cohort

Characteristics	HC (n = 21) (%)	Patient (n = 104)		
		CHB (n = 29) (%)	LC (n = 29) (%)	HCC (n = 46) (%)
Age (year)				
Mean ± SD	49.4 ± 10.8	36.6 ± 8.6	46.6 ± 7.0	54.4 ± 8.6
Sex				
Male	18(85.7)	26 (89.7)	25 (86.2)	41 (89.1)
Female	3 (14.3)	3 (10.3)	4 (13.8)	5 (10.9)
HBsAg				
Yes	\	29 (100)	29 (100)	46 (100)
No	\	0	0	0
Cirrhosis				
Yes	\	\	29 (100)	32 (69.56)
No	\	\	0	14 (30.44)
Child-Pugh				
A	\	\	6 (20.7)	46 (100)
B	\	\	12 (41.4)	0
C	\	\	11 (37.9)	0
TNM Stage				
I	\	\	\	18 (39.1)
II	\	\	\	15 (32.6)
III	\	\	\	12 (26.1)
IV	\	\	\	1 (2.2)

HBsAg, Hepatitis B surface antigen; TNM, Tumor-Node-Metastasis (TNM) staging system.

**Table 2:** Patient baseline characteristics of the validation cohort

Characteristics	HC (n = 15) (%)	Patient (n = 60)		
		CHB (n = 15) (%)	LC (n = 15) (%)	HCC (n = 30) (%)
Age (year)				
Mean ± SD	43.9 ± 11.9	43.3 ± 8.5	49.2 ± 7.1	56.5 ± 10.6
Sex				
Male	13 (86.7)	13 (86.7)	13 (86.7)	26 (86.7)
Female	2 (13.3)	2 (13.3)	2 (13.3)	4 (13.3)
HBsAg				
Yes	\	15 (100)	29 (100)	30 (100)
No	\	0	0	0
Child-Pugh				
A	\	\	5 (33.3)	20 (66.7)
B	\	\	5 (33.3)	9 (30)
C	\	\	5 (33.3)	1 (3.3)
TNM Stage				
I	\	\	\	10 (33.3)
II	\	\	\	10 (33.3)
III	\	\	\	10 (33.3)

HBsAg, Hepatitis B surface antigen.

**Table 3:** Patient baseline characteristics of the additional cohort

Characteristics	HC (n = 20) (%)	Patient (n = 110)		
		CHB (n = 25) (%)	LC (n = 30) (%)	HCC (n = 45) (%)
Age (year)				
Mean ± SD	50.0 ± 14.1	46.3 ± 10.9	49 ± 10.12	54.0 ± 10.0
Sex				
Male	14 (70.0)	15 (60.0)	18 (60.0)	28 (62.2)
Female	6 (30.0)	10 (40.0)	12 (40.0)	17 (37.8)
HBsAg				
Yes	\	25 (100)	30 (100)	45 (100)
No	\	0	0	0

HBsAg, Hepatitis B surface antigen.

### Fabrication of Antibody Microarrays

The antibody microarray that detects 532 antibodies (*e.g.*, cytokines, chemokines) was designed and fabricated as previously described (18). Briefly, all antibodies (Bio-Techne Ltd) (Abcam) were printed onto a 3D modified glass slide surface (Capital Biochip Corp) in duplicate at a concentration of 0.2 mg/ml using an Arrayjet microarrayer (Roslin) (supplemental Table S1). Positive controls were Alexa Fluor 555 goat antihuman immunoglobulin G (10 μg/ml) and biotinylated human immunoglobulin G (100 μg/ml), while PBS and bovine serum albumin (100 μg/ml) (Sigma-Aldrich) were used as negative controls. One slide could detect 532 protein targets in four serum samples simultaneously.

### Measurement of the Serum Proteome Using Antibody Microarrays

The principle and workflow of the antibody microarray to detect serological proteins were described in our previous work (18). First, all samples were randomly numbered. Then, 10 μl from each serum sample were labeled with 1 μl of NHS-PEG4-Biotin (20 g/L in dimethyl sulfoxide) (Thermo Fisher Scientific) after a 10-fold dilution with 1 × PBS (pH 7.4). A Bio-Spin column (Bio-Rad) was then used to remove the excess biotin *via* centrifugation at 1000*g*. This procedure was repeated four times with 500 μl of 1 × PBS. The final flow-through fraction was diluted with 400 μl of 5% milk (w/v).

The antibody microarray was equilibrated to room temperature and blocked with 5% milk (w/v) for 1 h using an incubation tray. After removing the blocking buffer with a vacuum pump, the microarray was incubated with the precollected biotinylated serum for 2 h at room temperature. After that, the slide was washed with PBS + 0.05% Tween 20 three times. Next, 2 μg/ml streptavidin phycoerythrin (Thermo Fisher Scientific) was added to bind the captured biotinylated serum protein molecules on the slide. After washing three times, the slide was scanned with the GenePix 4000A microarray scanner (Molecular Devices), and the fluorescence images and results were exported using the GenePix Pro 7 image analysis software (Molecular Devices) (https://www.moleculardevices.com/products/additional-products/genepix-microarray-systems-scanners). Serological proteins that bound to the microarray (*i.e.*, “positive signal”) had a fluorescent signal that was at least the average signal of the negative controls (PBS) plus two SDs (18).

### Experimental Design and Statistical Rationale

A total of 200 serum samples in the discovery cohort (HC=21, CHB=29, LC=29, HCC=46) and validation cohort (HC=15, CHB=15, LC=15, HCC=30) were measured using DIA-MS. To evaluate the reproducibility of DIA-MS, the same tryptic-digested human HEK293T cell lysate was analyzed at 15 different time points throughout the period of experiments; the interassay *r* correlation ranged from 0.93 to 0.97. We created a multidisease spectral library using 100 serum samples obtained from five patient groups, including healthy controls (n = 20), Bechet's disease (n = 20), non–small cell lung cancer (n = 20), and liver diseases (n = 20). The multidisease spectral library included a total of 9104 precursors and 1254 proteins.

An additional cohort comprised of 120 serum samples (HC=20, CHB=25, LC=30, HCC=45) were measured using parallel reaction monitoring (PRM) to validate the selected proteins. According to previously published guidelines, the type of PRM analysis that was used in our study was a Tier 3 assay (23). Skyline (version 19.1) was used for data analysis (24). The DIA and PRM data have been deposited to the ProteomeXchange Consortium (http://proteomecentral.proteomexchange.org) *via* the iProX partner repository, with the dataset identifier PXD034201 (supplemental Table S2).

### Serum Sample Preparation for MS

The serum samples were centrifuged at 10,000 rpm for 3 min. Then, 2 μl of the serum supernatant was transferred to a 1.5 ml centrifuge tube, and the proteins were denatured with 100 μl of 6 M urea (Sigma-Aldrich). The disulfide reduction was performed for 60 min in a water bath at 37 °C with 1 μl of 1 M dithiothreitol and then alkylated with 10 μl of 500 mM iodoacetamide at 25 °C for 45 min in the dark. The solution was transferred to an Amicon Ultra centrifugal filter unit (0.5 ml, 30 K, Millipore) to precipitate the alkylated protein in the filter tube at 12,000 g. After washing three times with 200 μl of 50 mM NH_4_HCO_3_ (Sigma-Aldrich) at 12,000 g, the proteins were digested with 0.04 mg/ml trypsin at 37 °C for 16 h. The tryptic peptides were centrifuged for 15 min at 12,000*g*. The collected peptide solution was dried under vacuum and dissolved in 20 μl of 0.1% formic acid. The peptide concentration was determined with a DS-11 Spectrophotometer (DeNovix) at an absorbance of *A*_280_ nm.

### Generation of the Spectral Library

To construct the spectral library, a mixture of 100 μg of peptides from each disease samples was separated into ten fractions using a RIGOL L-3000 HPLC system (Puyuan Jingdian Science and Technology, Ltd). Then, the peptides mixture was injected into a Gemini-NX C18 110 Å column (250 × 4.6 mm, 5 μm particles, Phenomenex) at a flow rate of 1 ml/min using mobile phase A (2% acetonitrile [ACN], pH = 10) and mobile phase B (98% ACN, pH = 10). The gradient was set as follows: 5% to 30% B for 0 to 15 min, 30% to 80% B for 15 to 18 min, 80% B for 18 to 20 min, 80% to 2% B for 2 to 2.1 min, and then 2% B for 20.1 to 25 min.

Data-dependent acquisition (DDA) was performed with the Q Exactive HF Hybrid Quadrupole Orbitrap mass spectrometer (Thermo Fisher Scientific). Briefly, 3 μg peptides were loaded onto the C18 trap column (100 μm × 2 cm, self-packed) on the EASY-nLC 1200 System (Thermo Fisher Scientific) at a maximum pressure of 280 bar with 12 μl solvent A (0.1% formic acid), followed by isolation on an analytical column (150 μm × 250 mm, 1.9 μm 200 Å C18 particles) at a flow rate of 600 nl/min. A 120-min gradient was performed as follows: 7% to 15% solvent B (80% ACN, 0.1% formic acid) for 15 min, 15% to 30% solvent B for 75 min, 30% to 50% solvent B for 25 min, 50% to 95% solvent B for 2 min, and then 95% solvent B for 8 min.

The full MS1 scans were acquired from a range of 300 to 1400 *m/z* with a resolution of 60,000. The top 20 precursor ions were selected for MS2 by higher energy C-trap dissociation fragmentation at a normalized collision energy of 30 with a resolution of 15,000. The automatic gain control was set to 3e6 for full MS1 and 5e4 for MS2, with maximum ion injection times of 80 and 120 ms, respectively (supplemental Table S3).

### DIA of the Serum Proteome

The DIA analysis was performed with the same LC system condition of DDA. The DIA acquisition scheme consisted of 45 variable windows ranging from 350 to 1400 *m/z* with an overlap of 1 Da using the Q Exactive HF Hybrid Quadrupole Orbitrap mass spectrometer (Thermo Fisher Scientific). The sequential precursor isolation window setup was as follows: 374-412, 412-436.5, 436.5-457, 457-471.5, 471.5-483.5, 483.5-494.5, 494.5-507, 507-520.5, 520.5-533.5, 533.5-545, 545-554.5, 554.5-563.5, 563.5-573.5, 573.5-583.5, 583.5-593.5, 593.5-604, 604-615, 615-626, 626-636, 636-646, 646-657, 675-668.5, 668.5-680, 680-691, 691-702, 702-714, 714-726.5, 726.5-739.5, 739.5-753, 753-767, 767-781, 781-796, 796-812, 812-828.5, 828.5-846.5, 846.5-866, 866-887, 887-910, 910-935.5, 935.5-964, 964-998, 998-1040.5, 1040.5-1101, and 1101-1269 *m/z*. The DIA parameter was as follows: normalized collision energy was 28, total cycle time was 3.6 s, resolution was 30,000, and the automatic gain control was set to 1e6 with maximum ion injection times of 45 ms (supplemental Table S4).

### Methods for DIA Data Analysis

The identification and quantification of the DIA data were analyzed using the Spectronaut Pulsar 14 (https://biognosys.com/software/spectronaut/) (Biognosys) as previously described (25). Default settings were used unless otherwise noted. For identification, the DDA raw files were searched against the human SWISS-PROT database (20,412 entries, downloaded on January 12, 2019, from UniProt) to generate a spectral library using the BGS factory setting. The false discovery rate (FDR) was set to 1% at protein and peptide precursor levels, while peptides represented by 3 to 6 fragments were included in the spectral library. The iRT Calibration R square was set as 0.8. Finally, a multidisease spectral library was created containing 1254 proteins and 9104 precursors. The DDA raw data and multidisease spectral library file were deposited to the ProteomeXchange Consortium (http://proteomecentral.proteomexchange.org) *via* the iProX partner repository with the identifier PXD040603.

For quantification, DIA raw data were searched against the multidisease spectral library *via* the Spectronaut Pulsar 14. The iRT regression type was set as local (nonlinear) regression. Every peptide contained at least three fragment ions. The results were filtered using a Q value of 0.01 (FDR of 1%). The *p* value was determined using the Kernel Density Estimator. The DIA raw data and the Spectronaut searching file (.sne) were deposited into the iProX database with the identifier PXD034201 (supplemental Tables S6 and S7).

### PRM of Target Proteins

PRM of the target proteins was performed with the Orbitrap Fusion mass analyzer (Thermo Fisher Scientific). Briefly, 0.5 μg peptides were loaded onto a C18 trap column (100 μm × 2 cm, self-packed) on the EASY-nLC 1200 System (Thermo Fisher Scientific) at a maximum pressure of 280 bar with 12 μl solvent A, followed by isolation on an analytical column (150 μm × 250 mm, 1.9 μm 200 Å C18 particles) at a flow rate of 600 nl/min. The gradient was set as follows: 7% to 12% solvent B for 5 min, 12% to 30% solvent B for 40 min, 40% to 45% solvent B for 5 min, 45% to 95% solvent B for 2 min, and then 95% solvent B for 8 min.

PRM method development and optimization of target proteins were performed using Skyline (version 19.1) with a method, duration of 60 min (26). For the MS OT mode, the resolution was 120,000; the scan range was 400 to 1000 (*m/z*); and the maximum injection time was 50 ms. The tMS2 OT collision energy was 30% with an isolation window of 1.6, resolution of 30,000, scan range of 200 to 1600, and a maximum injection time of 54 ms (supplemental Table S5).

### Methods for PRM Data Analysis

Preparing the isolation list and developing the method for PRM analyses were based on identified proteins and validated using Skyline. The human-reviewed proteome database remained as a reference background proteome, and a library was prepared using the MS2 data obtained from the .pdResult from PD2.4. The proteins’ UniProt ID was used as an input list. The isolation list, which was filtered with unique peptide sequences with 6 to 25 amino acids and two missed cleavage, was then fed into the PRM method. As a result, 51 proteins with 210 peptides were scheduled (supplemental Table S8). Furthermore, the peak areas of the peptides in the PRM raw files were analyzed using Skyline (supplemental Table S9). The PRM raw data and Skyline document were also deposited to the iProX partner repository with the dataset identifier PXD034201.

### Bioinformatics Analysis

The functional annotation of serum proteins was performed with PANTHER (http://www.pantherdb.org/). The relationship between proteins and different diseases was analyzed by DisGeNET (https://www.disgenet.org/) (27). The biological processes analysis was performed by ClueGO of Cytoscape version 3.8 with a *p*-value cut-off <0.01 (28). Signaling pathways were analyzed by String version 11.5 (https://string-db.org/) (29). The hierarchical clustering analysis was performed by Morpheus (https://software.broadinstitute.org/morpheus/). The cluster trend analysis of the DEPs was performed using the Gene cluster trend version v0.1.0 in Hiplot (https://hiplot.com.cn/) (30). The tissue specificity and intracellular location information of the proteins were retrieved from the Human Protein Atlas database (https://www.proteinatlas.org/). Protein–protein network analysis of proteins in the multimarker panels was performed using Wu Kong's platform (https://www.omicsolution.com/wkomics/main/).

### Statistical Analysis

For the antibody microarray data, the averaged pixel intensity across the two technical replicates on the antibody array was used to represent the bound protein. Per array block, the lowest positive value was used to calculate “nonsignal” values. Buffer was used as the benchmark value for intersample normalization and considered as “negative control.” Proteins with a median intensity less than the negative controls were not considered in the subsequent analyses. For the DIA-MS data, the proteins were further filtered so that the missing values of DIA-MS identified protein were less than 75%. The intersample data were normalized using quantile normalization (31, 32). The remaining missing values were replaced with the minimum of each sample (33, 34).

Normalized antibody microarray data and DIA-MS data were subjected to the Kruskal–Wallis H test (*p* value < 0.05, for all groups) and Wilcoxon rank sum test (*p* value < 0.05, pairwise) to identify the DEPs for each live disease *via* the Python sciPy package (v1.5.0). Principal component analysis (PCA) used data from pairwise comparisons of DEPs with the Python scikit-learn package (v0.23.1).

We performed feature selection *via* training LASSO regression models in a 5-fold cross validation test which was randomly repeated for 100 times. After training each model, the proteins with nonzero weights were collected into the candidate pool, where the proteins within the top 10% frequencies were finally selected as features to construct the machine learning models.

Classical machine learning models included the Ridge classifier, K-nearest neighbors classifier, Gaussian Naïve Bayes classifier, decision tree classifier, random forest classifier, and support vector machine classifier. They were tested on both the discovery cohort (n = 125) and independent validation cohort (n = 75), with F1-score and AUC as critical evaluation metrics. More specifically, we used a 5-fold cross-validation method on the discovery cohort to get five submodels based on the training folds and then combined the predicted scores of the test fold to obtain the “test score.” These models made predictions on the validation cohort, and the average of their predicted scores was considered the “validation score.” Data splitting, feature selection, model training, and evaluation were done *via* the Python scikit-learn package (v 0.23.1).

For the PRM data, the intensity of each peptide was quantified by averaging the peak AUC of the top three fragment ions (b and y), and the average intensity of all the confidently identified peptides was used to calculate the protein intensity (35). For each sample, the protein with maximum intensity was used for normalization (36). Normalized PRM data was subjected to the Kruskal–Wallis H test with an FDR <0.05 and Wilcoxon rank sum test (FDR < 0.05, pairwise) with a fold change >1.2 to identify the DEPs for each live disease *via* the Python sciPy package (v1.5.0).

## Results

### Landscape Mapping of Serum Proteomes in HBV-Related Liver Diseases Using the ID-Map Platform

**Fig. 1:**
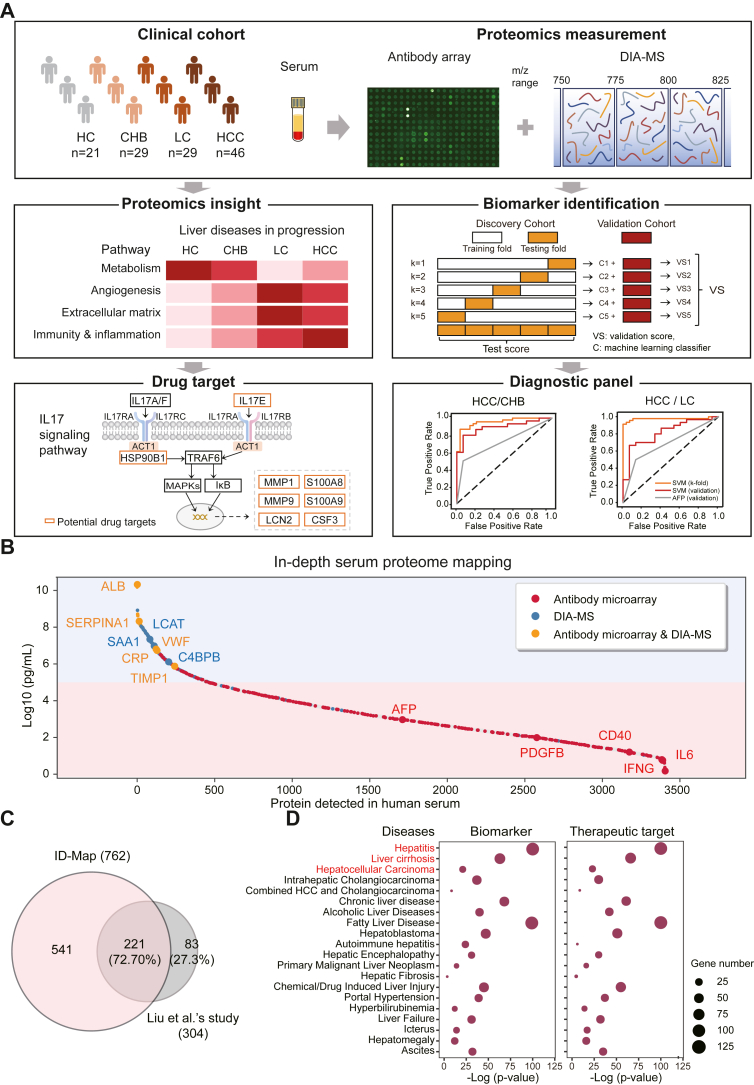
**Serum proteome analyses for liver disease patients using the in-depth serum proteome mapping platform.***A*, study design using in-depth serum proteome mapping (ID-Map). *B*, distribution of serum proteins detected by an antibody microarray and DIA-MS based on the reference concentrations provided in the human protein atlas (HPA) (https://www.proteinatlas.org/). *C*, a comparison of the proteins detected with the ID-Map platform and the proteins identified in published articles for four groups of serum samples (HC, CHB, LC, and HCC). *D*, bubble maps of liver diseases or their complications enriched in biomarkers and therapeutic targets through DisGeNET. CHB, chronic hepatitis B; DIA-MS, data-independent acquisition mass spectrometry; HC, healthy control; HCC, hepatocellular carcinoma; LC, liver cirrhosis.

The overall design of this study is shown in Figure 1*A*. We analyzed 125 proteomes in the serum of HCs (n = 21) and patients diagnosed with CHB (n = 29), LC (n = 29), or HCC (n = 46) using our ID-Map platform, which was comprised of a high-density antibody microarray and DIA-MS (supplemental Fig. S1 and Table 1). The results identified a total of 762 nonredundant proteins (antibody microarray: 525, DIA-MS: 365), which measured 541 more proteins than previous reports using the same disease cohort and constitutes the largest serum proteome database of liver diseases (CHB, LC, and HCC) to date (37) (Fig. 1*C*). The abundance distribution of these proteins in serum is ∼10 orders of magnitude according to the reference concentrations in the human plasma proteome database (http://www.plasmaproteomedatabase.org/) (supplemental Table S10) (18). Notably, the dataset includes many known proteins (IL6, IL10, IFNG, CSF3, PDGFB, CD40, and AFP) and novel proteins associated with liver diseases (Fig. 1*B*).

The reproducibility of the antibody microarray within and between different experiments was evaluated. The interassay and intraassay *r* correlations were 0.97 and 0.98, respectively (supplemental Fig. S2). The reproducibility of the DIA-MS method was determined by analyzing tryptically digested human HEK293T cell lysate throughout the period of experiments. The DIA-MS interassay *r* correlation ranged from 0.93 to 0.97 (supplemental Fig. S3*A*).

The enriched signaling pathways in the serum proteomes of patients with liver diseases included inflammation, blood coagulation, apoptosis, and angiogenesis (supplemental Fig. S4). The protein class analysis revealed that the serum proteins detected in this work belong to intercellular signaling molecules, defense/immunity enzymes, and metabolite interconversion enzymes (supplemental Fig. S5).

Of the total proteins detected, 408 and 377 serological proteins were recognized as potential biomarkers or drug targets in the PubMed database (https://pubmed.ncbi.nlm.nih.gov/) or Therapeutic Target Database (http://idrblab.net/ttd/) (38), respectively (supplemental Fig. S6 and supplemental Table S11). Notably, the proteins analyzed in this study are associated with hepatitis, LC, HCC, and alcoholic liver disease based on an enrichment analysis using DisGeNET (https://www.disgenet.org/) (Fig. 1*D* and supplemental Table S12). In view of our previous studies and the data obtained from this work, these results demonstrate the capability of our ID-Map platform in measuring serological proteins and its application in translational studies for HBV-related liver diseases (18, 20, 39).

### Biological Trajectory of Liver Diseases from Normal, CHB, LC to HCC

**Fig. 2:**
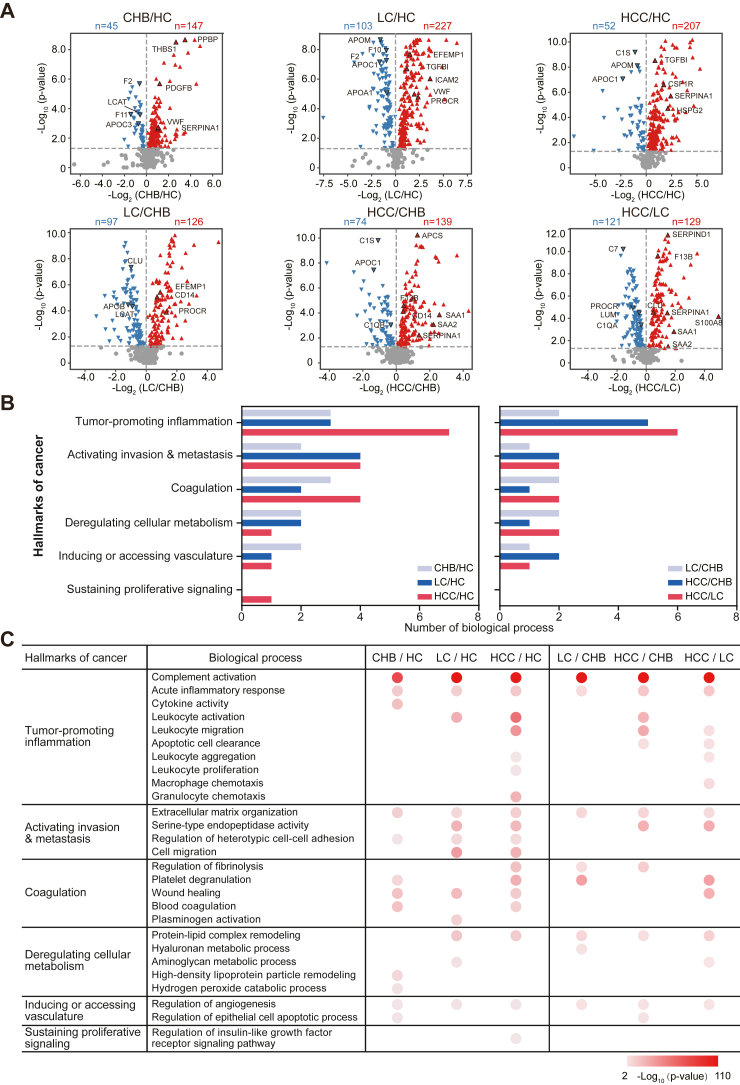
**Proteomics analysis of the biological trajectory of liver diseases from HCs, CHB, LC to HCC.***A*, identification of DEPs in the liver disease groups compared to HCs and to each other using volcano plot analysis. The selection of DEPs was performed using the Wilcoxon rank sum test analysis (*p* value < 0.05). *Blue* and *red dots* represent downregulated and upregulated proteins. *B*, the number and type of biological processes involved in the hallmarks of cancer that are enriched in the DEPs across the different patient groups. *C*, biological process analysis of DEPs in CHB *versus* HC, LC *versus* HC, HCC *versu*s HC, LC *versus* CHB, HCC *versus* CHB, and HCC *versus* LC using Cytoscape and ClueGo version 3.8. (*p* value < 0.01). The *light* to *dark red color* indicates the low to high significance of biological processes, respectively. CHB, chronic hepatitis B; DEP, differentially expressed protein; HCC, hepatocellular carcinoma; HC, healthy control; LC, liver cirrhosis.

Using the Wilcoxon rank sum test (*p* < 0.05), 192, 330, and 259 DEPs were identified by comparing the liver diseases (CHB, LC, and HCC) to the HC group, respectively (Fig. 2*A* and supplemental Fig. S7; supplemental Table S13). PCA analysis of all these proteins revealed that the HC, LC, and HCC groups were distinct from each other but CHB and HCC were not (supplemental Fig. S8). Therefore, we created the PCA analysis for all three diseases and HC group using the corresponding pairwise DEPs, demonstrating the capability of these DEPs in discriminating between HBV-related liver diseases (supplemental Fig. S9).

Using ClueGO in Cytoscape, our results revealed the cancerous trajectory of liver diseases from HC, CHB, LC to HCC, in which the majority of biological processes that changed in CHB, LC, and HCC diseases were discovered to be hallmarks of cancer, including inflammation, metastasis, metabolism, and vasculature (40) (Fig. 2*B*). Notably, coagulation was also identified as an additional hallmark of cancer that is exclusively present in blood since it is closely associated with the initiation, progression, and prognosis of different cancers (41).

The altered biological processes that are involved in tumor-promoting inflammation, activating invasion and metastasis, coagulation, and sustaining proliferative signaling continually increased with liver disease progression (*i.e.*, from CHB to LC to HCC) (Fig. 2*B*). The CHB patients had altered complement, acute phase response, cytokine activity, extracellular matrix organization, regulation of heterotypic cell-cell adhesion, endothelial cell proliferation, and wound healing (Fig. 2*C*). In comparison, the LC patients had altered biological processes that included leukocyte activation and protein–lipid complex remodeling, aminoglycan metabolic process, cell migration, and serine-type endopeptidase activity. Finally, more immune signaling (*i.e.*, leukocyte aggregation, migration, proliferation, and granulocyte chemotaxis) and sustained proliferative signaling (*i.e.*, regulation of insulin-like growth factor receptor signaling pathway) were activated in HCC patients. These data constitute a serum proteome landscape that represents the hallmarks of cancer in liver disease patients with HBV infection. The results are supported by previous studies in which the integration of HBV infection led to host chromosome instability and promoted cancer development, metastasis, and angiogenesis by regulating the telomerase reverse transcriptase, tumor protein 53, catenin beta 1, and other proteins associated with tumor development (42, 43). Notably, cytokine–cytokine receptor interaction and viral protein interaction were ranked as the top dysregulated signaling pathways in all patient groups in this study, indicating the central importance of inflammation throughout liver disease with HBV infection (supplemental Fig. S10) (44, 45, 46, 47, 48).

The results are also consistent with the DEPs that are dysregulated in HCC patients compared to CHB and LC patients because the DEPs were enriched in the biological processes of hallmarks of cancer, including tumor-promoting inflammation, activating invasion and metastasis, and coagulation (Fig. 2, *A*–*C*). The data suggest that different HBV-related liver diseases alter different biological processes.

### Consistent Clustering of Serological Proteins Based on Liver Disease Progression

**Fig. 3:**
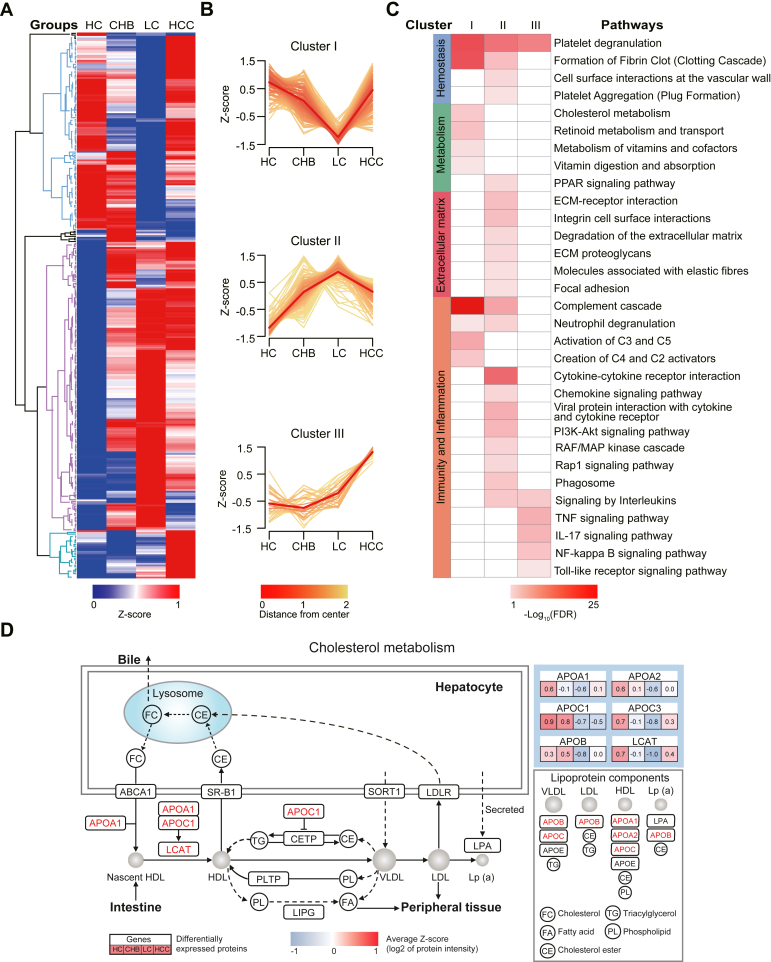
**Hierarchical clustering analyses of serum proteomes in liver diseases.***A*, hierarchical clustering map of the DEPs identified in HC, CHB, LC, and HCC patients (*p* value < 0.05). A false color scheme from *blue* to *red* represents the minimum and maximum Z-score values, respectively. *B*, proteins clustered into three groups according to their expression patterns, and the Z-scores were plotted over four groups using the gene cluster trend of Hiplot. *C*, pathway analysis of the DEPs was performed per cluster using the STRING database (version 11.5.). The false discovery rate (FDR) value indicates the significance of pathways, where a lower FDR represents a higher significance. *D*, the DEPs involved in cholesterol metabolism are summarized by the average Z-score across four groups (HC, CHB, LC, and HCC). CHB, chronic hepatitis B; DEP, differentially expressed protein; HCC, hepatocellular carcinoma; HC, healthy control; LC, liver cirrhosis.

To understand the association between serological proteins and liver disease progression, hierarchical clustering for all DEPs in HCs and liver disease groups was performed, with which three clusters (I–III) were generated (Fig. 3, *A* and *B*; supplemental Fig. S11; supplemental Table S14). Proteins in “cluster I” were continually expressed at lower levels from HCs to CHB patients to LC patients and then increased in HCC patients. These DEPs were significantly enriched in pathways involved in immunity and inflammation (*e.g.*, complement cascade, neutrophil degranulation, activation of C3 and C5, and creation of C4 and C2 activators), metabolism (*e.g.*, cholesterol metabolism, retinoid and vitamin metabolism, metabolism of vitamins and cofactors, and vitamin digestion and absorption), and hemostasis (*e.g.*, platelet degranulation and formation of fibrin clot) (Fig. 3*C*).

Proteins in “cluster II,” however, had a protein expression pattern that was the exact opposite of those in “cluster I:” continually increased levels from HCs to CHB patients to LC patients and then decreased in HCC patients. The DEPs in “cluster II” were significantly enriched in immune and inflammatory pathways (*e.g.*, complement cascade, cytokine-cytokine receptor interaction, chemokine signaling pathway, PI3K-Akt, RAF/MAP kinase cascade, Rap1 signaling pathway, and focal adhesion), extracellular matrix (ECM)-related (*e.g.*, ECM-receptor interaction, ECM-proteoglycans, and molecules associated with elastic fibers), and hemostasis (*e.g.*, platelet degranulation, formation of fibrin clot, cell surface interactions at the vascular wall, and platelet aggregation).

Notably, the expression of proteins in “cluster III” continuously increased from HCs to CHB patients to LC patients and, finally, to HCC patients. Immune and inflammatory signaling pathways were enriched, including those regulated by interleukins, TNF, IL-17, NF-kappa B, and Toll-like receptor pathways.

Of the signaling pathways enriched across the three clusters identified with hierarchical clustering, cholesterol metabolism is of particular interest due to its association with viral infection, replication, and assembly (49). In this work, serological proteins enriched in cholesterol metabolism continually decreased in expression from HCs to LC patients and then increased in HCC patients (Fig. 3*D*). These proteins include lecithin-cholesterol acyltransferase (LCAT) and apolipoproteins (APOA1, APOA2, APOC1, APOC3, and APOB), which are involved in lipoprotein synthesis, transportation, and transformation. The differential expression of these cholesterol-associated proteins might be due to the enhanced consumption of cholesterols by HBV-infected host cells in CHB and LC patients. In HCC patients, the proliferation, migration, and metastasis of cancer cells may increase cholesterol metabolism. Indeed, cholesterol metabolism is upregulated in the tissue of HCC patients (32). In addition, O-acyltransferase 1 (SOAT1) is upregulated in HCC patients with a poor prognosis, whereas inhibiting SOAT1 significantly reduces the size of tumors when SOAT1 expression is high (32).

### Identification of Potential Drug Targets for Liver Disease Treatment

**Fig. 4:**
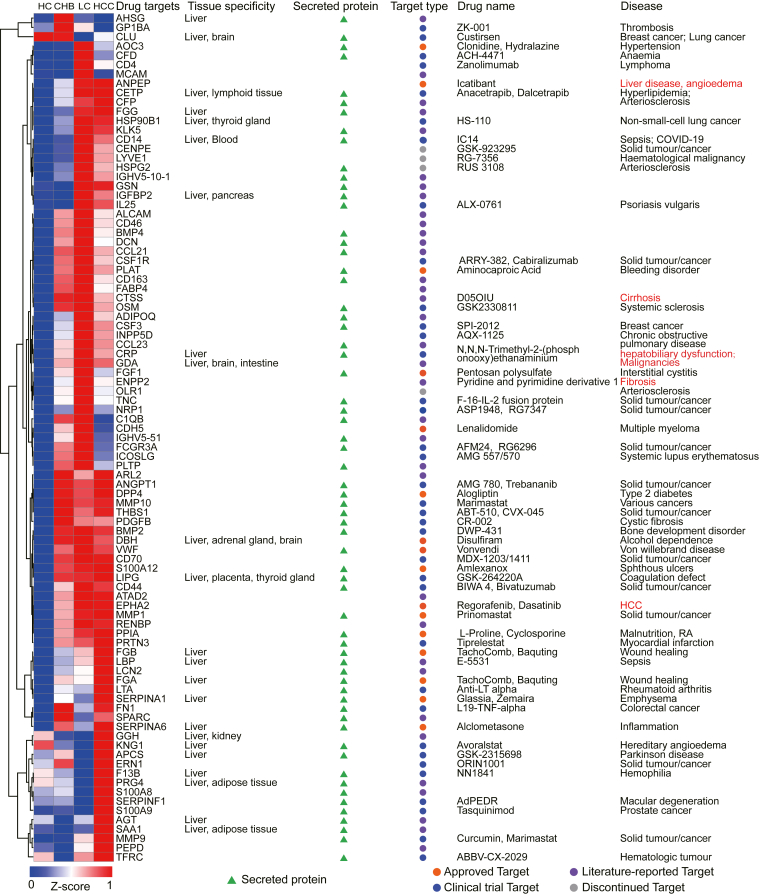
**Potential therapeutic targets of liver diseases identified by in-depth serum proteomics according to the Therapeutic Target Database.** Ninety-one drug targets were identified by cross-referencing the upregulated proteins in the three liver disease groups (CHB, LC, and HCC) discovered in this study with the Therapeutic Target Database (TTD) database. Tissue specificity and cellular location were obtained from the HPA database, while the target type, drug name, and disease were obtained from the TTD database. A false color scheme from *blue* to *red* represents the minimum and maximum Z-score values, respectively. CHB, chronic hepatitis B; HCC, hepatocellular carcinoma; HPA, human protein atlas; LC, liver cirrhosis.

**Fig. 5:**
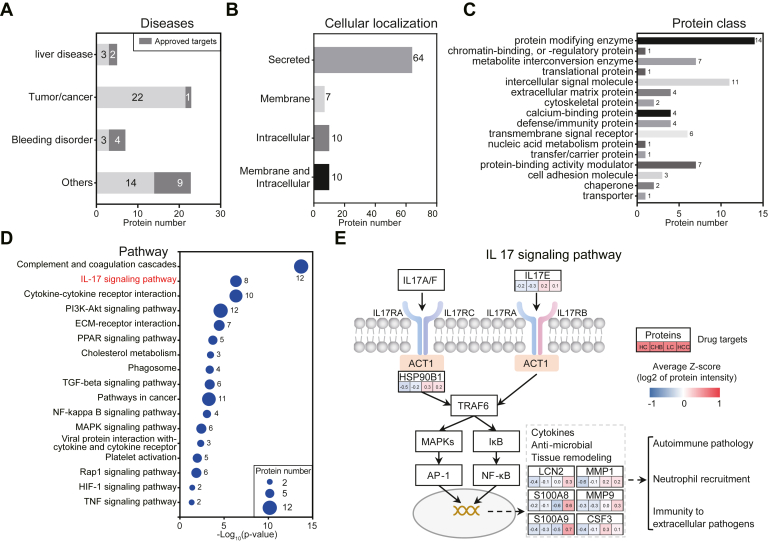
**Functional analyses of potential drug targets for liver diseases.***A*, the diseases treated by drugs that target one of 91 proteins according to the TTD. *B*, the cellular localization of the potential drug targets was obtained from the HPA database (https://www.proteinatlas.org/). *C*, protein classes of the potential drug targets were identified using PANTHER (http://www.pantherdb.org/). *D*, enriched signaling pathways in liver disease-related therapeutic targets based on information obtained from DisGeNET. The size of the *blue circle* represents the number of DEPs in the pathways. *E*, therapeutic targets involved in the IL17 signaling pathway. Means of the Z-score were used to represent the alterations in HC, CHB, LC, and HCC. CHB, chronic hepatitis B; DEP, differentially expressed protein; HC, healthy control; HCC, hepatocellular carcinoma; HPA, human protein atlas; LC, liver cirrhosis; TDD, Therapeutic Target Database.

Two hundred eighty-two proteins were upregulated (*p* < 0.05) in patient groups with liver diseases (CHB, LC, or HCC) compared to the healthy controls (Fig. 2*A* and supplemental Table S13). To better understand the potential of serum proteomics in finding therapeutic targets for liver diseases, these proteins were cross referenced to drug targets in the Therapeutic Target Database (http://idrblab.net/ttd/) (50); 91 proteins were identified as drug targets (Fig. 4 and supplemental Table S7). Of them, five proteins are targeted by drugs to treat liver diseases. For example, ANPEP is a target of the drug, Icatibant, which is used to treat refractory ascites in patients with LC. The pyridine and pyrimidine derivative 1 drugs that target ENPP2 are used for treating fibrosis. D05OIU, which is used to treat cirrhosis, targets CTSS. N, N, N-Trimethyl-2-(phosphonoxy) ethanaminium that targets C-reactive protein is used for treating hepatobiliary dysfunction and malignancies. Regorafenib and Dasatinib drugs target Ephrin type-A receptor 2 and are approved to treat HCC (51, 52, 53, 54). The 91 proteins also include 22 proteins, seven proteins, and 23 proteins that are drug targets for treating different cancers, bleeding disorders, or other diseases, respectively (Figs. 4 and 5A).

Functional annotation of the 91 proteins indicated that 25.27% (23/91) of them are produced in the liver, 70.33% (64/91) are secreted proteins, and 29.67% (27/91) are membrane or intracellular proteins (Fig. 5*B*). Protein class analysis showed that these proteins are protein-modifying enzymes, intercellular signal molecules, transmembrane signal receptors, or protein-binding activity modulators (Fig. 5*C*). The proteins are also involved in a variety of different cancer signaling pathways, including IL17, TNF, NF-kappa B, PI3K-Akt, TGF-beta, MAPK, Rap1, and HIF-1 signaling pathways (Fig. 5*D*). Interestingly, the IL17 signaling pathway is involved in inflammation, which is associated with all three liver diseases. Also, five proteins (HSP90B1, Protein S100-A9 [S100A9], MMP1, matrix metalloproteinase-9 [MMP9], and CSF3) in the IL17 signaling pathway are in phase III clinical trials for treating solid tumors and have the potential to treat liver diseases (Fig. 5*E* and supplemental Table S15).

### Protein Biomarker Signature for Diagnosing LC and HCC Patients

**Fig. 6:**
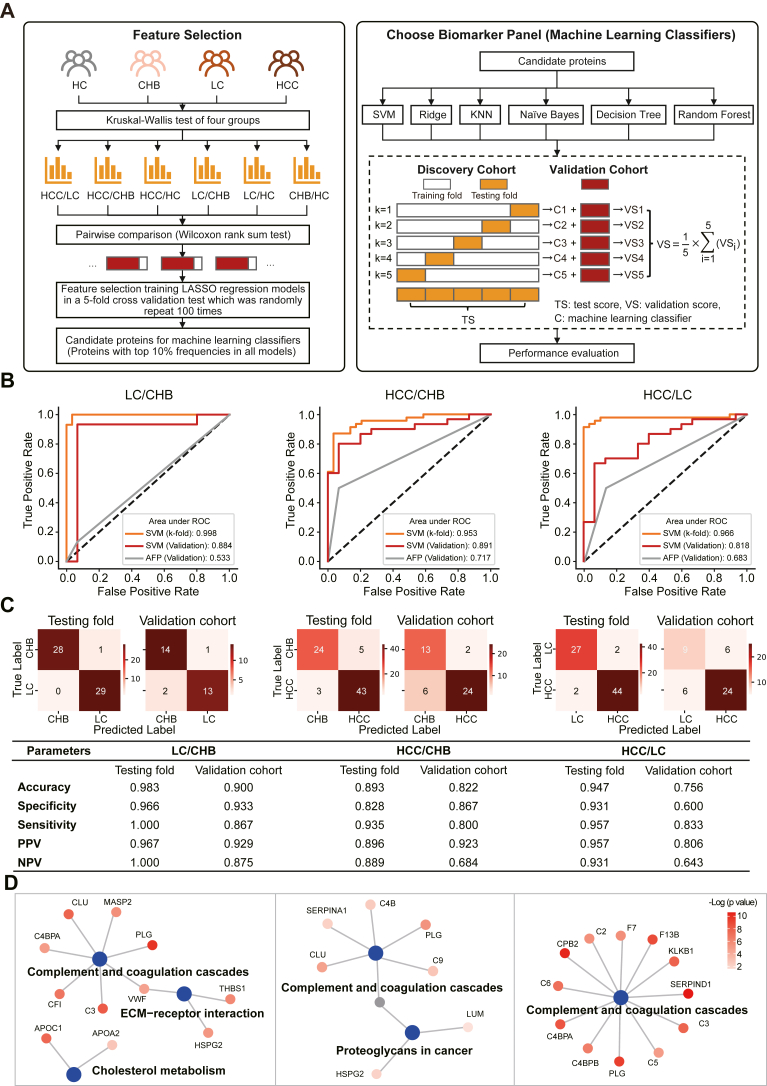
**Development of serum protein signatures differentiating liver diseases using machine learning.***A*, workflow of feature selection and machine learning modeling. *B* and *C*, the receiver operating characteristic (ROC) curve (*B*) and confusion matrix performance (*C*) of biomarker panels in LC *versus* CHB, HCC *versus* CHB, HCC *versus* LC of the discovery cohort and validation cohort. *D*, protein-protein network analysis of proteins in the multimarker panels of LC *versus* CHB, HCC *versus* CHB, and HCC *versus* LC. CHB, chronic hepatitis B; HCC, hepatocellular carcinoma; LC, liver cirrhosis.

To validate the candidate biomarkers detected in the discovery cohort (n = 125), the proteins were measured with DIA-MS using an independent cohort of 75 patients (supplemental Fig. S3*B* and Table 2). Three hundred thirteen proteins were reproducibly detected in both cohorts (supplemental Fig. S12). By training LASSO regression models in a 5-fold cross validation test that was randomly repeated for 100 times, multibiomarker panels to detect liver diseases were identified by feature selection (Fig. 6A and supplemental Table S16). We then compared six advanced machine learning classifiers and determined a support vector machine model as the final classifier for its overall outstanding performance (supplemental Fig. S13 and supplemental Table S17). Panel performances were quantified using AUCs and metrics derived from the confusion matrix for pairwise comparison of HCs and CHB, LC, and HCC patient groups (Fig. 6, *B* and *C*; supplemental Fig. S14).

Using machine learning, six multimarker panels were obtained to classify each liver disease (CHB, LC, HCC) from HCs as well as from each other (*i.e.*, LC *versus* CHB, HCC *versus* CHB, HCC *versus* LC) (supplemental Table S16). Compared to the single biomarker, AFP, the assay performance of multimarker panels for the detection of LC and HCC patients in high-risk CHB and/or LC patient groups are significantly improved. For example, the AUCs for detecting LC in CHB patients using a multibiomarker panel are 0.998 and 0.884 in the discovery cohort and validation cohort, respectively, whereas it is 0.533 when using AFP alone. Using multibiomarker panels, the AUC for detecting HCC in CHB and LC patients are 0.953 and 0.966 in the discovery cohort and 0.891 and 0.818 in the validation cohort, respectively, while it is 0.717 and 0.683 when using AFP alone (Fig. 6, *B* and *C*; supplemental Table S17).

The protein–protein network analysis revealed that the biomarker proteins in these panels were enriched in pathways related to liver diseases, including complement and coagulation cascades, ECM–receptor interaction, cholesterol metabolism, and proteoglycans in cancer (Fig. 6*D*). Therefore, the panels identified by our serum proteomics platform provide a resource of candidate biomarkers for diagnosing LC and HCC.

Of the biomarker panels identified in this study (supplemental Table S16), we confirmed the known biomarkers of LC (*i.e.*, ICAM2, LUM, and LGALS3BP) and HCC (*i.e.*, SERPINA1, CLU, A2M, IGFBP2, VWF, FUCA1, and FBLN1) (supplemental Fig. S15*A* and supplemental Table S18). In addition, many novel liver disease biomarkers were discovered, such as EGF-containing fibulin-like extracellular matrix protein 1 (EFEMP1), SAA2, CPN2, ANPEP, TGFBI, and FGG (supplemental Fig. S15*B*). For example, it has been reported that the level of mRNA that encodes for the EFEMP1 significantly correlates with fibrosis in nonalcoholic fatty liver disease patients, while the EFEMP1 protein can inhibit the proliferation, migration, and apoptosis of HCC cells (55, 56). For the first time, we have associated EFEMP1 as a protein biomarker of HBV-related LC. Moreover, SAA is an acute-phase protein family, and we observed that SAA1, SAA2, and SAA4 protein levels increased significantly as cancer progressed (57).

Fifty-one proteins of each panel in the additional cohort (n = 120) were measured using an orthogonal platform, PRM. Twenty-three proteins were validated, including known biomarkers (*e.g.*, VWF, PPBP) and newly identified biomarkers of HCC (*e.g.*, ANPEP, PIGR, AFM) and LC (*e.g.*, CPN2) (supplemental Fig. S16; Table 3 and supplemental Table S19).

## Discussion

In this work, we analyzed the serological proteins of patients with liver diseases that represent disease progression, from HCs to CHB to LC and, finally, to HCC. A total of 762 proteins were measured, which constitutes the largest proteomics dataset from patients with different liver diseases to date (Fig. 1, *B* and *C*). Notably, using bioinformatics analysis, we further reveal the trajectory of the hallmarks of cancer in the serum of CHB, LC, and HCC patients with HBV infection.

Interestingly, the hierarchical clustering of DEPs revealed three protein clusters according to the changes in their expression from HCs to CHB to LC to HCC (Fig. 3, *A* and *B*). Serological proteins in cluster I were enriched in signaling pathways involved in coagulation, metabolism, and the immune system. For example, in cholesterol metabolism, the expression of LCAT and apolipoproteins (APOA1, APOA2, APOC1, APOC3, and APOB) that are involved in cholesterol metabolism continually decreased from HCs to LC patients and then increased in HCC patients (Fig. 3*D*). The results are in accordance with a previous study, which showed that cholesterol metabolism was dysregulated in HCC tumor tissue when compared to neighboring noncancer tissue using genomics and proteomics tools (32). In addition, APOA1 and APOA2 are the main components of high-density lipoprotein, while APOB is the main component of low-density lipoproteins (LDLs) and very low-density lipoproteins. LCAT converts free cholesterol in serum into cholesterol esters that can be stored in high-density lipoprotein and then transported to the liver for further metabolism (58). APOB is the recognition site of the LDL receptor on the plasma membrane, and a functional study using human HepG2 cells showed that enhanced cholesterol metabolism in hepatocytes stimulates the secretion of APOB and reduces the uptake of LDL (59). Notably, all cholesterol metabolism proteins identified in this work are synthesized in the liver and secreted into the blood. The upregulation of these proteins in HCC patients may indicate abnormal hypermetabolism in HCC cells (32). Mipomersen, an antisense oligonucleotide inhibitor of APOB synthesis, is approved by the U. S. Food and Drug Administration as an orphan drug for use in familial hypercholesterolemia (60). Our data indicate that it may also have the potential to be used in HCC treatment (supplemental Fig. S11*A*).

In contrast to cluster I, the serological proteins in cluster II displayed the exact opposite expression profile. Furthermore, the proteins were enriched in ECM, inflammation, and angiogenesis pathways (Fig. 3*C*). Indeed, the upregulation of profibrotic proteins in LC and HCC patients that belong to the ECM (HSPG2, LUM, FBLN1, TNXB, TNC, and EFEMP1), inflammation (IL3, CXCL14, CCL21, BMP2, BMP4, OSM, and TIMP1), and angiogenesis (VWF, ANGPT1, protein C receptor [PROCR]) has been previously reported (61, 62, 63, 64). The results are consistent with hepatic fibrosis characteristics and provide abundant information on the mechanism and treatment of liver diseases (65, 66). For example, silymarin and glycyrrhizic acid are drugs used to treat LC in the clinic. Silymarin achieves antifibrosis effects by inhibiting the activation of hepatic stellate cells to reduce the generation of ECM, while downregulating metalloproteinase inhibitor 1 (TIMP1) that can enhance collagen degradation (67, 68). Glycyrrhizic acid has anti-inflammatory and hepatoprotective effects by inhibiting the activation of PI3K-Akt and MAPK pathways (69, 70, 71). These disrupted pathways were also identified in this work (Fig. 3*C*). Notably, endothelial PROCR, which is specifically overexpressed in LC patients, meditates angiogenesis by activating the PI3K–Akt signaling pathway. As such, PROCR may serve as a new drug target for LC patients (72) (supplemental Fig. S11*B*).

Proteins in cluster III are of great interest because their expression continually increases as the severity of the liver disease increases. The proteins are enriched in IL-17, TNF, NF-κB, and Toll-like receptor signaling pathways (Fig. 3*C*). The results highlight the importance of inflammation in the progression of liver diseases from CHB to HCC (73). It is well known that HCC is an inflammation-driven cancer, and the significance of IL17A in the proliferation and migration of HCC has been investigated in tissue and mouse models (74, 75, 76). Three serum proteins (S100A9, MMP9, and lipocalin-2) in the IL-17 pathway were identified (Fig. 5*E*). S100A9 and MMP9 are associated with a poor prognosis of HCC and can promote the growth and metastasis of HCC cells by activating the MAPK signaling pathway and epithelial-mesenchymal transformation, respectively (77, 78, 79, 80). Lipocalin-2 is secreted mainly by HCC cells into the bloodstream, and some data suggest that it can promote the invasion and metastasis of HCC through the Met–FAK axis (81, 82, 83) (supplemental Fig. S11*C*).

There remains a clinical need to better detect HCC since AFP has limited specificity and sensitivity (84, 85, 86). The need to detect HCC in CHB and LC patients is particularly important because they are at high risk of developing HCC. As the earliest diagnostic biomarker of HCC, AFP has limited sensitivity and specificity (84, 85, 86). Moreover, AFP levels are normal in 40% of HCC patients, while also being elevated in patients with chronic hepatitis, LC, and other cancers (87, 88, 89, 90). Although more biomarkers have been identified that may improve HCC detection when combined with AFP, the sensitivity and specificity remain unsatisfactory (10, 11). In this work, we used machine learning to develop multimarker panels that discriminate HCC from CHB and HCC from LC with AUCs of 0.891 and 0.818, respectively, which are significantly higher than using AFP alone. The results indicate the potential of using biomarkers to diagnose HCC in high-risk populations. However, it should be noted that the number of clinical serum samples can influence results and statistical analysis. As such, these candidate biomarkers should be validated in a larger, independent cohort in the future.

## Conclusion

In this work, an in-depth analysis of serum proteomes in HCs and patients with progressing liver diseases (CHB, LC, and HCC) was performed using antibody microarrays and mass spectrometry. Our results provide fundamental insights into the changes of the serum proteome during liver disease progression. In addition, we identified proteins that may be effective diagnostic biomarkers or therapeutic drug targets for liver diseases. Lastly, this translational serology-based approach could be used to study other diseases.

## Data Availability

The DIA and PRM data have been deposited to the ProteomeXchange Consortium (http://proteomecentral.proteomexchange.org) *via* the iProX partner repository with the dataset identifier PXD034201 (91, 92). The DDA data were deposited as a separate submission *vi*a the iProX partner repository with the dataset identifier PXD040603.

## Supplemental Data

This article contains supplemental data.

## Conflict of interest

The authors declare they have no competing interests.
