# Supplementary material for: In-Depth Serum Proteomics Reveals the Trajectory of Hallmarks of Cancer in Hepatitis B Virus–Related Liver Diseases
Source: Mol Cell Proteomics. 2023 May 19;22(7):100574. doi: 10.1016/j.mcpro.2023.100574 (PMC10316086; doi:10.1016/j.mcpro.2023.100574)
Supplement: Supplemental Table S3 [file mmc3.docx]

**Method of DDA**

**OVERALL METHOD SETTINGS**

Global Settings

Use lock masses best

Chrom. peak width (FWHM) 25 s

Time

Method duration 120.00 min

Dynamic Exclusion 20.0 ppm

**Experiment**

**FULL MS / DD-MS² (TOPN)**

**General**

Runtime 0 to 120 min

Polarity Positive

In-source CID 0.0 eV

Default charge state 3

**Full MS**

Microscans 1

Resolution 60,000

AGC target 3e6

Maximum IT 80 ms

Number of scan ranges 1

Scan range 300 to 1400 m/z

Spectrum data type Centroid

**dd-MS² / dd-SIM**

Microscans 1

Resolution 15,000

AGC target 5e4

Maximum IT 120 ms

Loop count 20

MSX count 1

TopN 20

Isolation window 1.6 m/z

Isolation offset 0.0 m/z

Scan range 200 to 2000 m/z

Fixed first mass 120.0 m/z

(N)CE / stepped (N)CE nce: 30

Spectrum data type Centroid

**dd Settings**

Minimum AGC target 1.00e3

Intensity threshold 8.3e3

Charge exclusion unassigned, 1, 7, 8, >8

Peptide match Preferred

Exclude isotopes on

Dynamic exclusion 20.0 s

**Setup**

**CONTACT CLOSURE**

**General**

Used False

Start in Closed True

Switch Count 0

**SYRINGE**

**General**

Used False

Start in OFF True

Stop at end of run False

Switch Count 0

Pump setup

Syringe type Hamilton

Flow rate 3.000 µL/min

Inner diameter 2.303 mm

Volume 250 µL

**DIVERT VALVE A**

**General**

Used False

Start in 1-2 True

Switch Count 0

**DIVERT VALVE B**

**General**

Used False

Start in 1-2 True

Switch Count 0
