# Supplementary material for: In-Depth Serum Proteomics Reveals the Trajectory of Hallmarks of Cancer in Hepatitis B Virus–Related Liver Diseases
Source: Mol Cell Proteomics. 2023 May 19;22(7):100574. doi: 10.1016/j.mcpro.2023.100574 (PMC10316086; doi:10.1016/j.mcpro.2023.100574)
Supplement: Supplemental Table S4 [file mmc4.docx]

**Method of DIA**

**OVERALL METHOD SETTINGS**

Use lock masses off

Chrom. peak width (FWHM) 30 s

Method duration 120.00 min

Dynamic Exclusion 20.0 ppm

**FULL MS — SIM**

**General**

Runtime 0 to 120 min

Polarity Positive

In-source CID 0.0 eV

**Setting**

Microscans 1

Resolution 60,000

AGC target 3e6

Maximum IT 80 ms

Number of scan ranges 1

Scan range 350 to 1400 m/z

Spectrum data type Centroid

**DIA**

**General**

Runtime 0 to 120 min

Polarity Positive

In-source CID 0.0 eV

Default charge state 2

**DIA**

**Setting**

Microscans 1

Resolution 30,000

AGC target 1e6

Maximum IT 45 ms

(N)CE / stepped (N)CE nce: 28

Spectrum data type Centroid

Overlap 1Da

Cycle time 3.6s

**INCLUSION LIST**

45 entries

Mass Formula Species CS Polarity Start End (N)CE MSX ID Comment

[m/z] [M] [z] [min] [min]

374.00000 2 Positive 8.00 115.00 28

554.50000 2 Positive 8.00 115.00 28

563.50000 2 Positive 8.00 115.00 28

583.50000 2 Positive 8.00 115.00 28

545.00000 2 Positive 8.00 115.00 28

604.00000 2 Positive 8.00 115.00 28

626.00000 2 Positive 8.00 115.00 28

636.00000 2 Positive 8.00 115.00 28

646.00000 2 Positive 8.00 115.00 28

691.00000 2 Positive 8.00 115.00 28

483.50000 2 Positive 8.00 115.00 28

494.50000 2 Positive 8.00 115.00 28

573.50000 2 Positive 8.00 115.00 28

593.50000 2 Positive 8.00 115.00 28

668.50000 2 Positive 8.00 115.00 28

615.00000 2 Positive 8.00 115.00 28

657.00000 2 Positive 8.00 115.00 28

680.00000 2 Positive 8.00 115.00 28

702.00000 2 Positive 8.00 115.00 28

714.00000 2 Positive 8.00 115.00 28

471.50000 2 Positive 8.00 115.00 28

520.50000 2 Positive 8.00 115.00 28

533.50000 2 Positive 8.00 115.00 28

726.50000 2 Positive 8.00 115.00 28

739.50000 2 Positive 8.00 115.00 28

507.00000 2 Positive 8.00 115.00 28

753.00000 2 Positive 8.00 115.00 28

767.00000 2 Positive 8.00 115.00 28

781.00000 2 Positive 8.00 115.00 28

457.00000 2 Positive 8.00 115.00 28

796.00000 2 Positive 8.00 115.00 28

812.00000 2 Positive 8.00 115.00 28

828.50000 2 Positive 8.00 115.00 28

846.50000 2 Positive 8.00 115.00 28

866.00000 2 Positive 8.00 115.00 28

887.00000 2 Positive 8.00 115.00 28

412.00000 2 Positive 8.00 115.00 28

910.00000 2 Positive 8.00 115.00 28

436.50000 2 Positive 8.00 115.00 28

935.50000 2 Positive 8.00 115.00 28

964.00000 2 Positive 8.00 115.00 28

998.00000 2 Positive 8.00 115.00 28

1040.50000 2 Positive 8.00 115.00 28

1101.00000 2 Positive 8.00 115.00 28

1269.00000 2 Positive 8.00 115.00 28

**EXCLUSION LIST**

(no entries)

**NEUTRAL LOSSES**

(no entries)

**MASS TAGS**

(no entries)
