# Supplementary material for: In-Depth Serum Proteomics Reveals the Trajectory of Hallmarks of Cancer in Hepatitis B Virus–Related Liver Diseases
Source: Mol Cell Proteomics. 2023 May 19;22(7):100574. doi: 10.1016/j.mcpro.2023.100574 (PMC10316086; doi:10.1016/j.mcpro.2023.100574)
Supplement: Supplemental Table S5 [file mmc5.docx]

**Method of PRM**

**OVERALL METHOD SETTINGS**

Use Ion Source Settings from Tune = False

Method Duration (min)= 60

Ion Source Type = NSI

Spray Voltage: Positive Ion (V) = 2200

Spray Voltage: Negative Ion (V) = 600

Sweep Gas (Arb) = 0

Ion Transfer Tube Temp (°C) = 320

APPI Lamp = Not in use

Pressure Mode = Standard

Default Charge State = 2

Advanced Precursor Determination = False

**Experiment 1**

Start Time (min) = 0

End Time (min) = 60

Cycle Time (sec) = 3

**Scan MasterScan**

MSn Level = 1

Use Wide Quad Isolation = True

Detector Type = Orbitrap

Orbitrap Resolution = 120K

Mass Range = Normal

Scan Range (m/z) = 400-1000

Maximum Injection Time (ms) = 50

AGC Target = 400000

Microscans = 1

RF Lens (%) = 60

Use ETD Internal Calibration = False

DataType = Profile

Polarity = Positive

Source Fragmentation = False

Scan Description =

**Experiment 1**

Start Time (min) = 0

End Time (min) = 60

Cycle Time (sec) = 3

**Scan tMSn**

MSn Level = 2

Isolation Mode = Quadrupole

Isolation Window = 1.6

Define MSX IDs = False

Multiplex Ions Enabled = False

Maximum number of multiplexed ions = 0

MS2 CID Activation Time (ms) = 10

Loop Count= 20

Loop Control= 3

Loop Time= 3

ActivationType = HCD

Collision Energy (%) = 30

Stepped Collision Energy (%) = 5

Is Stepped Collision Energy On = False

Detector Type = Orbitrap

Orbitrap Resolution = 30K

Mass Range = Normal

Scan Range (m/z) = 200-1600

Maximum Injection Time (ms) = 54

AGC Target = 50000

Inject ions for all available parallelizable time = True

Microscans = 1

Use ETD Internal Calibration = False

DataType = Centroid

Polarity = Positive

Source Fragmentation = False

Scan Description =

**>>>>>>>>>>>>> Mass List Table <<<<<<<<<<<<<<**

CompoundName Formula| AdductPositive| m/z| z| t start (min)| t stop (min)|

| | +H| 563.2916| 2| 12.85| 16.85|

| | +H| 706.7623| 2| 13.96| 17.96|

| | +H| 657.3203| 2| 16.78| 20.78|

| | +H| 438.5493| 3| 16.81| 20.81|

| | +H| 516.272| 2| 18.07| 22.07|

| | +H| 583.296| 2| 18.75| 22.75|

| | +H| 452.2427| 2| 18.78| 22.78|

| | +H| 411.7214| 2| 19.06| 23.06|

| | +H| 574.2798| 2| 19.22| 23.22|

| | +H| 562.7682| 2| 20.28| 24.28|

| | +H| 532.2762| 2| 20.48| 24.48|

| | +H| 494.7691| 2| 20.52| 24.52|

| | +H| 414.7449| 2| 21.3| 25.3|

| | +H| 431.8943| 3| 21.35| 25.35|

| | +H| 647.3379| 2| 21.36| 25.36|

| | +H| 526.7484| 2| 21.5| 25.5|

| | +H| 557.8144| 2| 21.71| 25.71|

| | +H| 419.7189| 2| 21.79| 25.79|

| | +H| 433.5544| 3| 21.91| 25.91|

| | +H| 649.828| 2| 21.93| 25.93|

| | +H| 503.2536| 2| 22.08| 26.08|

| | +H| 739.3313| 2| 22.44| 26.44|

| | +H| 511.7431| 2| 23.51| 27.51|

| | +H| 810.8843| 2| 23.55| 27.55|

| | +H| 540.9253| 3| 23.57| 27.57|

| | +H| 506.7873| 2| 23.7| 27.7|

| | +H| 468.8845| 3| 24.14| 28.14|

| | +H| 456.7158| 2| 24.16| 28.16|

| | +H| 527.7982| 2| 25.75| 29.75|

| | +H| 466.2766| 2| 25.78| 29.78|

| | +H| 444.7555| 2| 25.95| 29.95|

| | +H| 514.7904| 2| 26.71| 30.71|

| | +H| 484.798| 2| 26.73| 30.73|

| | +H| 738.3454| 2| 26.76| 30.76|

| | +H| 491.7876| 2| 26.89| 30.89|

| | +H| 577.757| 2| 27.09| 31.09|

| | +H| 560.8091| 2| 27.48| 31.48|

| | +H| 437.7427| 2| 27.49| 31.49|

| | +H| 610.327| 3| 28.1| 32.1|

| | +H| 914.9869| 2| 28.12| 32.12|

| | +H| 639.8563| 2| 28.23| 32.23|

| | +H| 456.7611| 2| 28.27| 32.27|

| | +H| 537.7749| 2| 28.43| 32.43|

| | +H| 586.6479| 3| 28.69| 32.69|

| | +H| 879.4683| 2| 28.71| 32.71|

| | +H| 443.2312| 4| 28.89| 32.89|

| | +H| 885.455| 2| 28.91| 32.91|

| | +H| 590.6391| 3| 28.92| 32.92|

| | +H| 525.2905| 2| 29.11| 33.11|

| | +H| 461.7533| 2| 29.21| 33.21|

| | +H| 644.8226| 2| 29.33| 33.33|

| | +H| 430.2175| 3| 29.37| 33.37|

| | +H| 678.8433| 2| 29.45| 33.45|

| | +H| 643.294| 2| 29.57| 33.57|

| | +H| 658.8535| 2| 29.61| 33.61|

| | +H| 439.5714| 3| 29.67| 33.67|

| | +H| 447.2456| 2| 29.68| 33.68|

| | +H| 523.9399| 3| 29.7| 33.7|

| | +H| 785.4063| 2| 29.72| 33.72|

| | +H| 474.7793| 2| 29.78| 33.78|

| | +H| 790.4094| 2| 29.89| 33.89|

| | +H| 527.2753| 3| 29.94| 33.94|

| | +H| 859.9467| 2| 30.09| 34.09|

| | +H| 573.6335| 3| 30.1| 34.1|

| | +H| 572.814| 2| 30.71| 34.71|

| | +H| 464.7585| 2| 30.79| 34.79|

| | +H| 511.7795| 2| 30.87| 34.87|

| | +H| 582.29| 3| 30.89| 34.89|

| | +H| 436.9693| 4| 30.89| 34.89|

| | +H| 872.9313| 2| 30.91| 34.91|

| | +H| 773.3538| 2| 31.2| 35.2|

| | +H| 662.3038| 2| 31.26| 35.26|

| | +H| 570.8166| 2| 31.55| 35.55|

| | +H| 520.7904| 2| 31.66| 35.66|

| | +H| 556.7666| 2| 31.85| 35.85|

| | +H| 492.2797| 2| 31.98| 35.98|

| | +H| 606.6493| 3| 32.19| 36.19|

| | +H| 909.4703| 2| 32.21| 36.21|

| | +H| 543.3193| 2| 32.22| 36.22|

| | +H| 538.803| 2| 32.62| 36.62|

| | +H| 579.2997| 4| 32.73| 36.73|

| | +H| 772.064| 3| 32.75| 36.75|

| | +H| 475.2243| 2| 32.85| 36.85|

| | +H| 814.4017| 2| 33.13| 37.13|

| | +H| 543.2703| 3| 33.14| 37.14|

| | +H| 644.8177| 2| 33.54| 37.54|

| | +H| 512.746| 2| 34.2| 38.2|

| | +H| 438.2529| 2| 34.65| 38.65|

| | +H| 610.3357| 2| 34.75| 38.75|

| | +H| 407.2262| 3| 34.75| 38.75|

| | +H| 626.3326| 2| 35| 39|

| | +H| 417.8908| 3| 35.03| 39.03|

| | +H| 598.803| 2| 35.52| 39.52|

| | +H| 510.2635| 2| 35.54| 39.54|

| | +H| 550.8192| 2| 35.54| 39.54|

| | +H| 503.782| 2| 35.54| 39.54|

| | +H| 742.3614| 3| 35.9| 39.9|

| | +H| 557.0228| 4| 35.93| 39.93|

| | +H| 419.5342| 3| 36.34| 40.34|

| | +H| 628.7977| 2| 36.35| 40.35|

| | +H| 880.9354| 2| 36.51| 40.51|

| | +H| 565.0275| 4| 36.53| 40.53|

| | +H| 795.8992| 2| 36.56| 40.56|

| | +H| 530.9352| 3| 36.56| 40.56|

| | +H| 753.0343| 3| 36.57| 40.57|

| | +H| 540.2902| 2| 36.6| 40.6|

| | +H| 496.9029| 3| 36.78| 40.78|

| | +H| 744.8506| 2| 36.82| 40.82|

| | +H| 405.2049| 3| 36.84| 40.84|

| | +H| 607.3036| 2| 36.86| 40.86|

| | +H| 512.7966| 2| 36.88| 40.88|

| | +H| 555.8057| 2| 36.9| 40.9|

| | +H| 535.7487| 2| 36.98| 40.98|

| | +H| 500.0699| 5| 37.14| 41.14|

| | +H| 624.8356| 4| 37.15| 41.15|

| | +H| 832.7784| 3| 37.17| 41.17|

| | +H| 458.256| 2| 37.18| 41.18|

| | +H| 563.7562| 2| 37.61| 41.61|

| | +H| 808.3909| 2| 37.64| 41.64|

| | +H| 613.3086| 2| 37.72| 41.72|

| | +H| 424.726| 2| 37.73| 41.73|

| | +H| 550.2871| 2| 37.91| 41.91|

| | +H| 575.6161| 3| 38.01| 42.01|

| | +H| 862.9205| 2| 38.02| 42.02|

| | +H| 581.2735| 2| 38.36| 42.36|

| | +H| 622.3377| 2| 38.4| 42.4|

| | +H| 409.2502| 2| 38.45| 42.45|

| | +H| 867.4085| 2| 38.72| 42.72|

| | +H| 578.6081| 3| 38.72| 42.72|

| | +H| 584.2894| 2| 38.82| 42.82|

| | +H| 516.808| 2| 39.17| 43.17|

| | +H| 438.994| 4| 39.8| 43.8|

| | +H| 876.9808| 2| 39.81| 43.81|

| | +H| 584.9896| 3| 39.82| 43.82|

| | +H| 448.268| 2| 39.92| 43.92|

| | +H| 781.3677| 2| 40.08| 44.08|

| | +H| 521.2476| 3| 40.08| 44.08|

| | +H| 806.9257| 2| 40.19| 44.19|

| | +H| 538.2863| 3| 40.19| 44.19|

| | +H| 561.2866| 2| 40.96| 44.96|

| | +H| 508.3109| 2| 40.97| 44.97|

| | +H| 601.2798| 2| 40.98| 44.98|

| | +H| 446.8993| 3| 41.11| 45.11|

| | +H| 669.8453| 2| 41.12| 45.12|

| | +H| 414.5799| 3| 41.65| 45.65|

| | +H| 621.3663| 2| 41.66| 45.66|

| | +H| 794.8875| 2| 41.66| 45.66|

| | +H| 680.3497| 3| 41.7| 45.7|

| | +H| 530.2607| 3| 41.71| 45.71|

| | +H| 770.8676| 2| 42.48| 46.48|

| | +H| 514.2474| 3| 42.48| 46.48|

| | +H| 703.875| 2| 42.92| 46.92|

| | +H| 469.5858| 3| 42.94| 46.94|

| | +H| 481.2912| 2| 42.97| 46.97|

| | +H| 907.4414| 2| 43.27| 47.27|

| | +H| 605.2967| 3| 43.3| 47.3|

| | +H| 549.2595| 2| 43.7| 47.7|

| | +H| 522.6193| 3| 43.91| 47.91|

| | +H| 465.7452| 2| 44.21| 48.21|

| | +H| 634.6927| 3| 44.3| 48.3|

| | +H| 478.5663| 3| 44.3| 48.3|

| | +H| 717.3457| 2| 44.31| 48.31|

| | +H| 417.2311| 3| 44.36| 48.36|

| | +H| 625.343| 2| 44.37| 48.37|

| | +H| 951.5355| 2| 44.38| 48.38|

| | +H| 436.2842| 2| 44.9| 48.9|

| | +H| 512.7823| 2| 45.85| 49.85|

| | +H| 482.5587| 3| 46.32| 50.32|

| | +H| 723.3345| 2| 46.33| 50.33|

| | +H| 906.4785| 2| 46.69| 50.69|

| | +H| 604.6548| 3| 46.71| 50.71|

| | +H| 878.9363| 2| 47.72| 51.72|

| | +H| 586.2933| 3| 47.76| 51.76|

| | +H| 801.4359| 2| 48.24| 52.24|

| | +H| 534.6264| 3| 48.25| 52.25|

| | +H| 571.9805| 3| 48.9| 52.9|

| | +H| 857.4671| 2| 48.92| 52.92|

| | +H| 855.9623| 2| 49.3| 53.3|

| | +H| 742.3932| 2| 49.33| 53.33|

| | +H| 538.3235| 2| 49.6| 53.6|

| | +H| 850.9175| 2| 49.69| 53.69|

| | +H| 619.3159| 2| 50.38| 54.38|

| | +H| 747.7302| 3| 50.55| 54.55|

| | +H| 684.8433| 2| 50.57| 54.57|

| | +H| 862.9205| 2| 50.72| 54.72|

| | +H| 612.6548| 3| 50.72| 54.72|

| | +H| 459.7429| 4| 50.72| 54.72|

| | +H| 918.4785| 2| 50.73| 54.73|

| | +H| 587.619| 3| 50.88| 54.88|

| | +H| 598.6176| 3| 51.24| 55.24|

| | +H| 897.4227| 2| 51.28| 55.28|

| | +H| 728.3594| 2| 51.31| 55.31|

| | +H| 485.9087| 3| 51.32| 55.32|

| | +H| 681.3533| 3| 51.98| 55.98|

| | +H| 621.8765| 2| 52.23| 56.23|

| | +H| 414.9201| 3| 52.28| 56.28|

| | +H| 660.8438| 2| 52.3| 56.3|

| | +H| 617.7943| 2| 52.75| 56.75|

| | +H| 599.8164| 2| 53.2| 57.2|

| | +H| 671.3379| 2| 53.34| 57.34|

| | +H| 790.4927| 2| 54.85| 58.85|

| | +H| 686.897| 2| 55.35| 59.35|

| | +H| 558.2825| 3| 55.43| 59.43|

| | +H| 836.9201| 2| 55.46| 59.46|

| | +H| 771.378| 3| 55.59| 59.59|

| | +H| 905.4274| 2| 55.78| 59.78|

| | +H| 603.954| 3| 55.81| 59.81|

| | +H| 655.3611| 2| 55.94| 59.94|

| | +H| 622.3449| 3| 56| 59|

| | +H| 861.9567| 2| 56.3| 60|
