# Supplementary material for: In-Depth Serum Proteomics Reveals the Trajectory of Hallmarks of Cancer in Hepatitis B Virus–Related Liver Diseases
Source: Mol Cell Proteomics. 2023 May 19;22(7):100574. doi: 10.1016/j.mcpro.2023.100574 (PMC10316086; doi:10.1016/j.mcpro.2023.100574)
Supplement: Supplemental information [file mmc20.docx]

# Supplementary information

**In-depth serum proteomics reveals the trajectory of hallmarks of cancer in hepatitis B virus-related liver diseases**

Meng Xu^1, 2^*, Kaikun Xu^2, 8^*, Shangqi Yin^3^*, Wei Sun^2^*, Guibin Wang^2^, Kai Zhang^2^, Jinsong Mu^4^, Miantao Wu^5^, Baocai Xing^6^, Xiaomei Zhang^2^, Jinyu Han^3^, Xiaohang Zhao^7^, Cheng Chang^2, 8#^, Yajie Wang^3#^, Danke Xu^1#^ and Xiaobo Yu^2#^

1 State Key Laboratory of Analytical Chemistry for Life Science, School of Chemistry and Chemical Engineering, Nanjing University, Nanjing, China.

2 State Key Laboratory of Proteomics, Beijing Proteome Research Center, National Center for Protein Sciences, Beijing Institute of Lifeomics, Beijing, 102206, China.

3 Department of Clinical Laboratory, Beijing Ditan Hospital, Capital Medical University, Beijing 100015, China.

4 Department of Critical Care Medicine, The Fifth Medical Center, Chinese PLA General Hospital, Beijing, 100039, China

5 Sun Yat-sen University Cancer Center, State Key Laboratory of Oncology in South China, Collaborative Innovation Center for Cancer Medicine, Guangzhou 510060, China

6 Key Laboratory of Carcinogenesis and Translational Research (Ministry of Education/Beijing), Department of Hepato-Pancreato-Biliary Surgery I, Peking University Cancer Hospital and Institute, Beijing, 100036, China

7 State Key Laboratory of Molecular Oncology, Cancer Hospital, Chinese Academy of Medical Sciences and Peking Union Medical College, Beijing 100021, China

8 Research Unit of Proteomics Driven Cancer Precision Medicine, Chinese Academy of Medical Sciences, Beijing 102206, China.

* Contributed equally to this manuscript

## Supplemental Figures

| **Figure S1** | Display images of antibody microarrays detected with serum samples from HCs and HCC patients. |
| --- | --- |
| **Figure S2** | Reproducibility of serum detection using the antibody microarray. |
| **Figure S3** | Longitudinal quality control of mass spectrometry in the discovery cohort (A) and validation cohort (B) using a tryptic digest of human HEK293T cells. |
| **Figure S4** | Pathway analysis of 762 proteins detected by DIA-MS and antibody microarrays. |
| **Figure S5** | Protein class analysis of 762 proteins detected by DIA-MS and antibody microarrays. |
| **Figure S6** | Disease biomarkers or therapeutic targets of liver diseases (LC, CHB, HCC) in the PubMed database or Therapeutic Target Database (TTD). |
| **Figure S7** | The normal distribution histograms of DIA-MS data for all samples that before quantile normalization, after quantile normalization, and after replacing missing values. |
| **Figure S8** | Principal component analysis using DEPs identified between HCs and patients with liver diseases (CHB, LC and HCC). |
| **Figure S9** | Principal component analysis using DEPs identified between any two patient groups (HC, CHB, LC, HCC). |
| **Figure S10** | Signaling pathway analysis of DEPs between any two groups of samples (HC, CH B, LC, HCC) by the STRING database (p < 0.05). |
| **Figure S11** | The expression of representative proteins in cluster I (A), cluster II (B) and cluster III (C) in HC, CHB, LC, and HCC groups. |
| **Figure S12** | Venn diagram analysis of proteins detected in the discovery and validation cohorts using DIA-MS. |
| **Figure S13** | Comparison of the F1-score in six advanced machine learning classifiers (Support vector machine, Ridge, KNN, Naïve Bayers, Decision Tree and Random Forest). |
| **Figure S14** | The ROC curve (A) and confusion matrix performance (B) of biomarker panels in CHB versus HC, LC versus HC, and HCC versus HC across the discovery and validation cohorts. |
| **Figure S15** | Box plot analysis of protein biomarkers of HCC and LC that have been previously reported (A) and newly identified in this study (B). |
| **Figure S16** | Box plot analysis of representative biomarkers of CHB/HC, LC/HC, HCC/HC, LC/CHB and HCC/CHB in the additional cohort based on PRM data. |


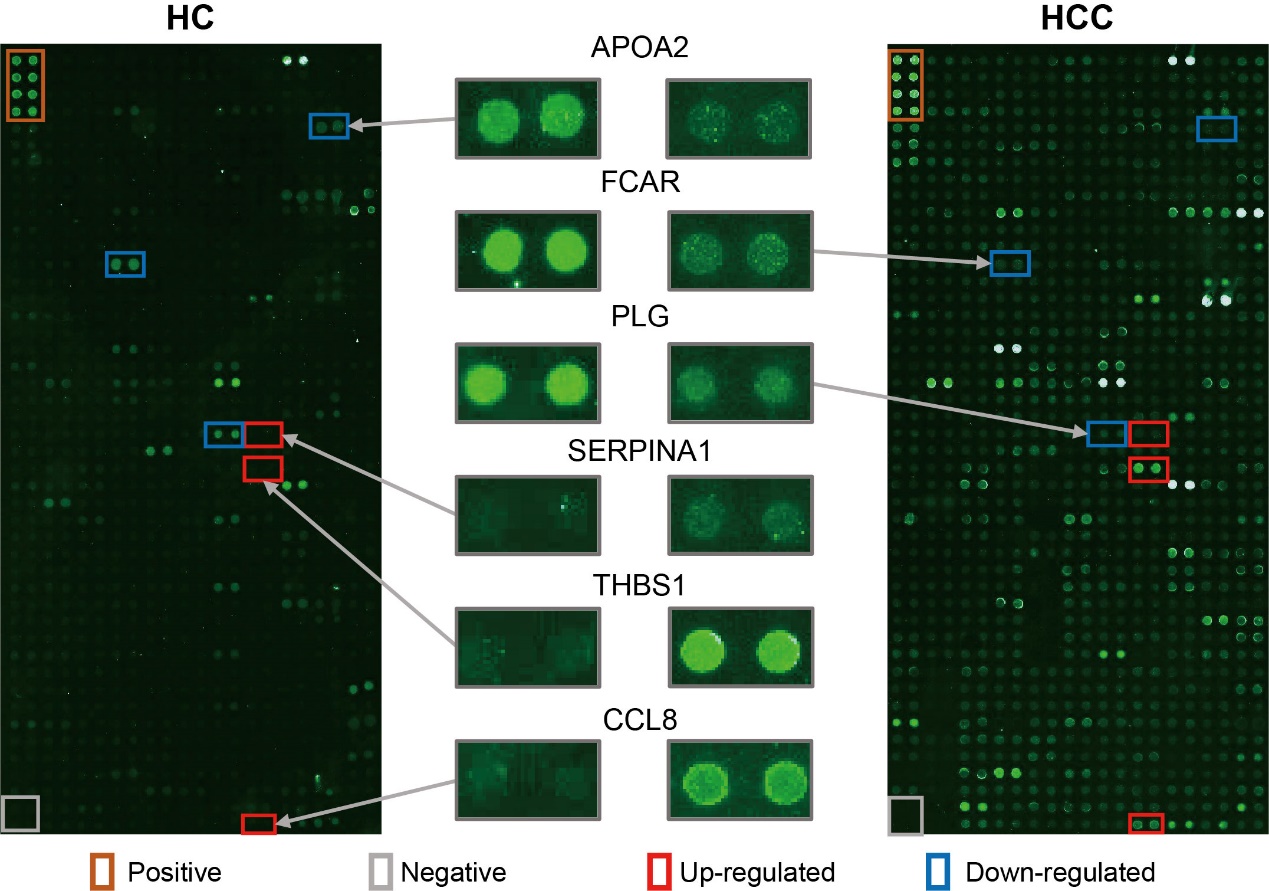


### Figure S1. Display images of antibody microarrays detected with serum samples from HCs and HCC patients.


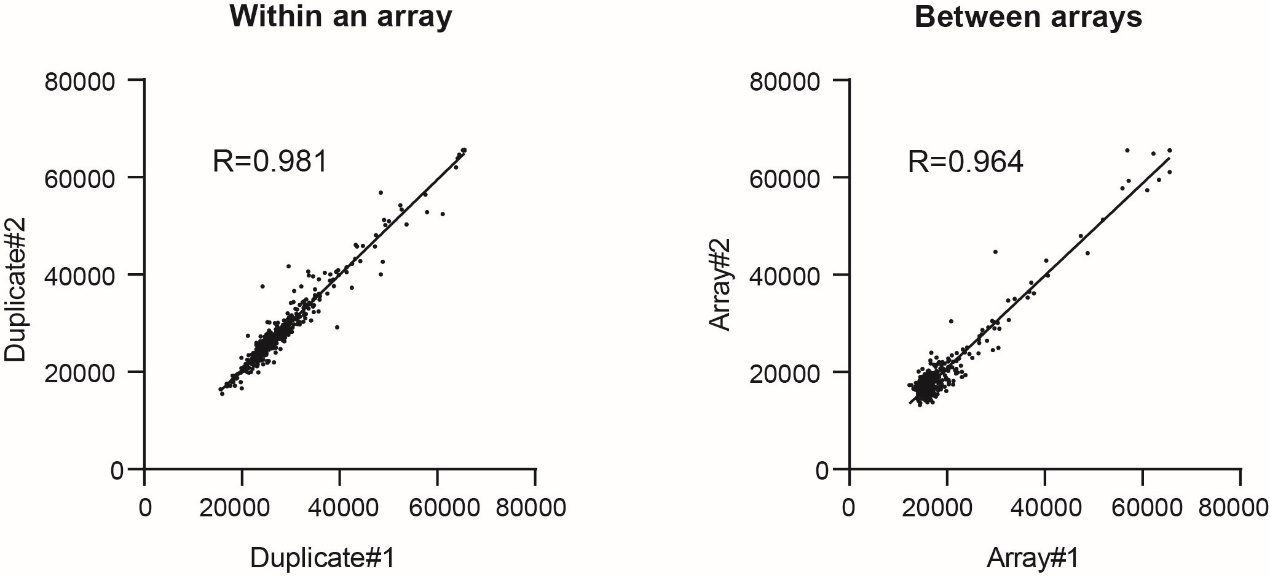


### Figure S2. Reproducibility of serum detection using the antibody microarray.


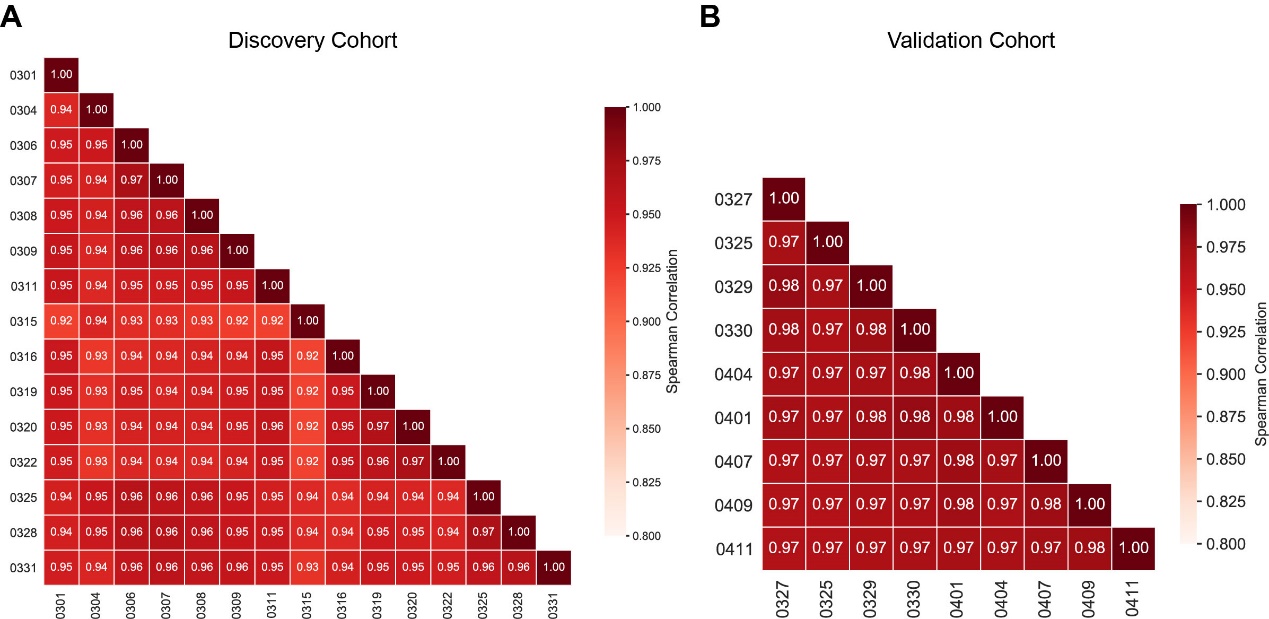


### Figure S3. Longitudinal quality control of mass spectrometry in the discovery cohort (A) and validation cohort (B) using a tryptic digest of human HEK293T cells. The bottom-left half of the panel represents the pairwise Spearman’s correlation coefficients of the samples.


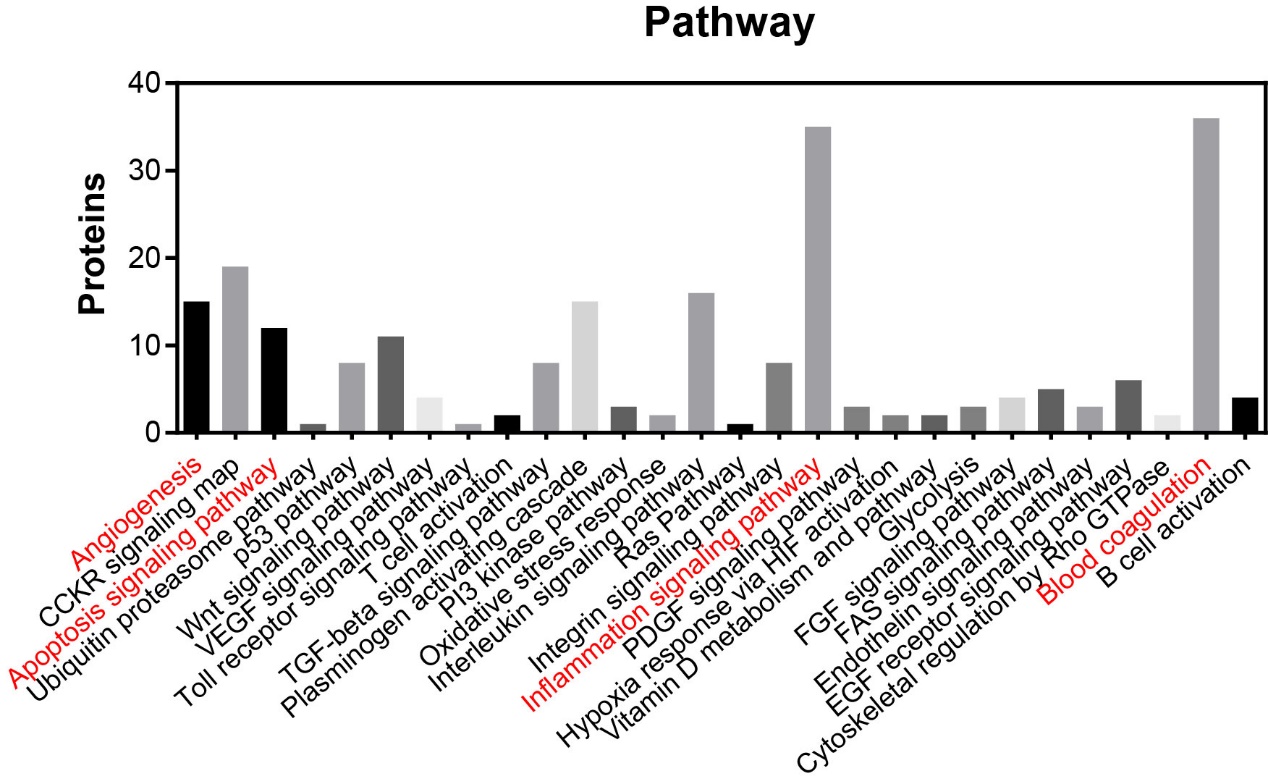


### Figure S4. Pathway analysis of 762 proteins detected by DIA-MS and antibody microarrays. Pathway analysis was performed using the PANTHER database (http://pantherdb.org/).


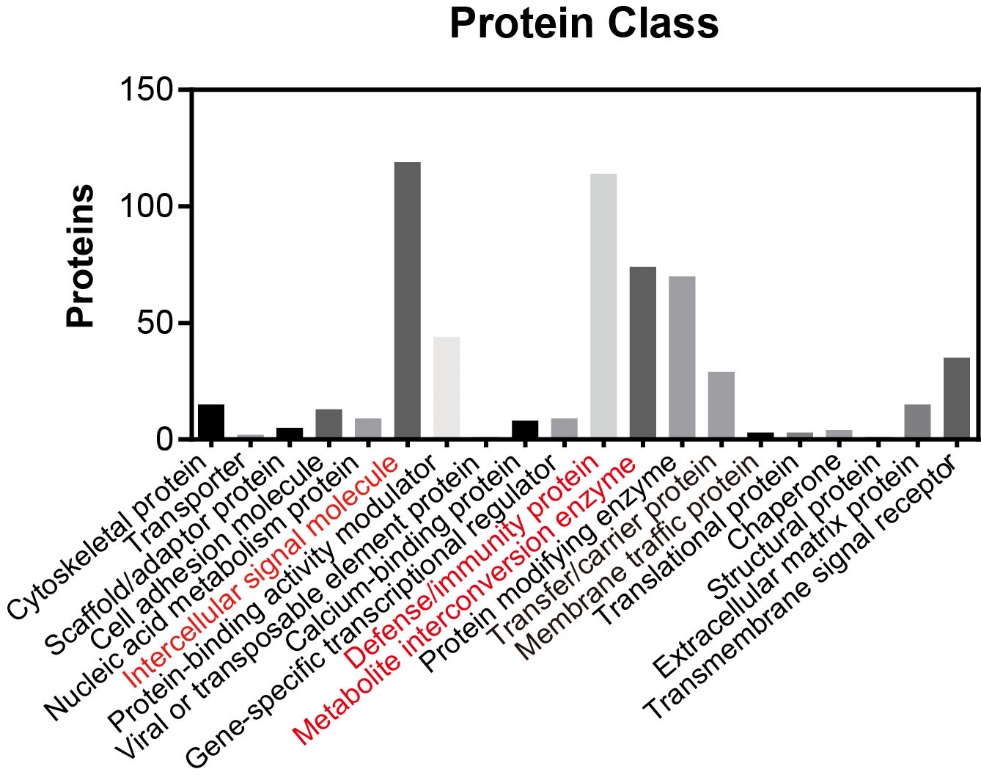


### Figure S5. Protein class analysis of 762 proteins detected by DIA-MS and antibody microarrays. Protein class analysis was performed using the PANTHER database (http://pantherdb.org/).


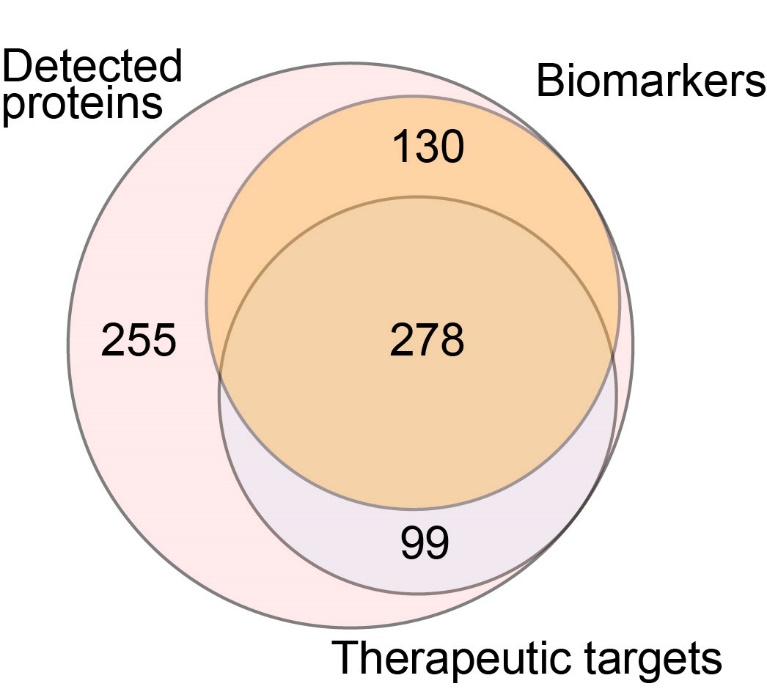


### Figure S6. Disease biomarkers or therapeutic targets of liver diseases (LC, CHB, HCC) in the PubMed database or Therapeutic Target Database (TTD).


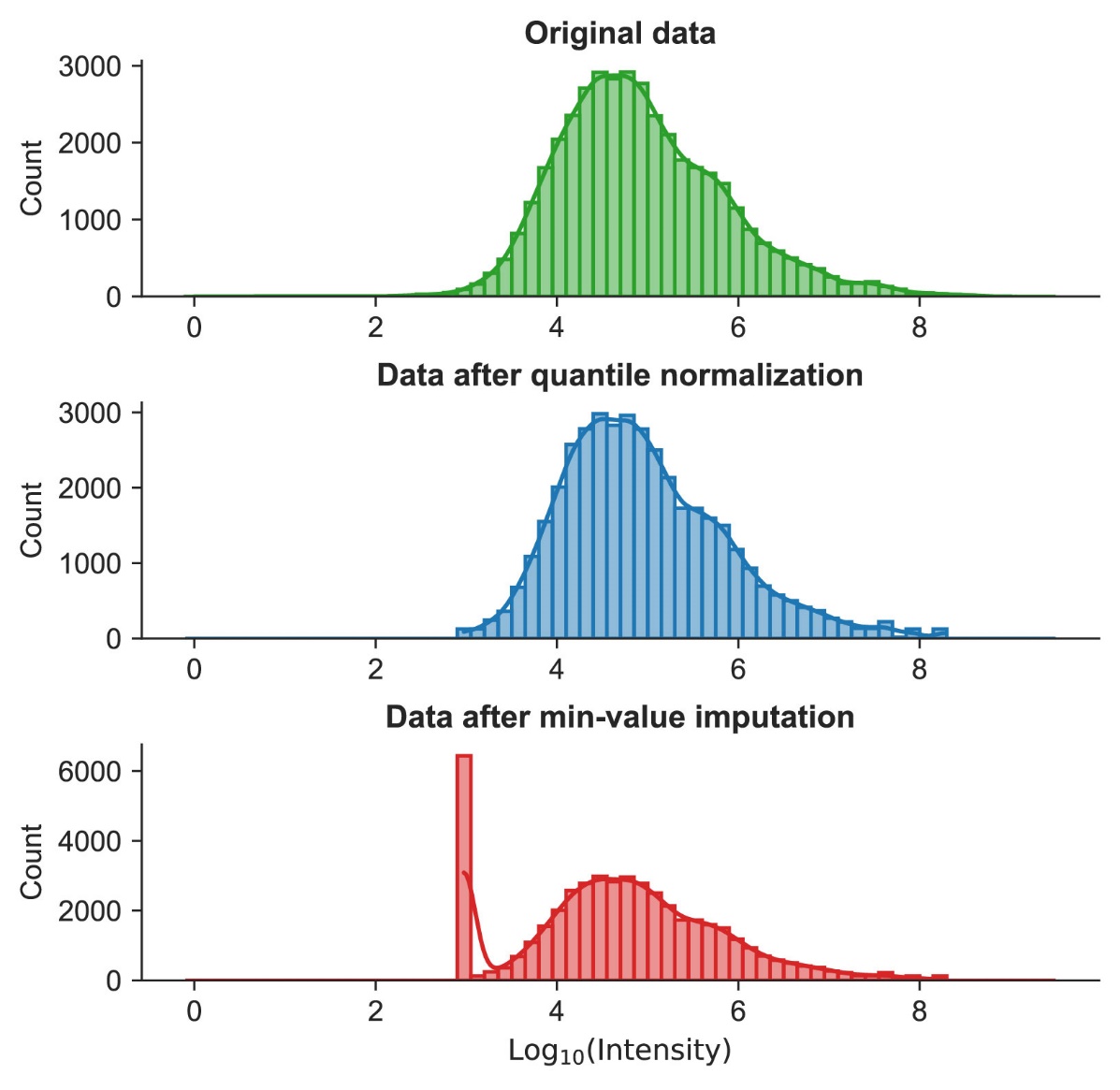


### Figure S7. The normal distribution histograms of DIA-MS data for all samples that before quantile normalization, after quantile normalization, and after replacing missing values.


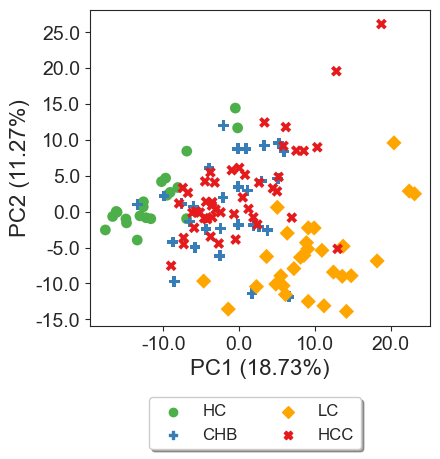


### Figure S8. Principal component analysis using DEPs identified between HCs and patients with liver diseases (CHB, LC and HCC).


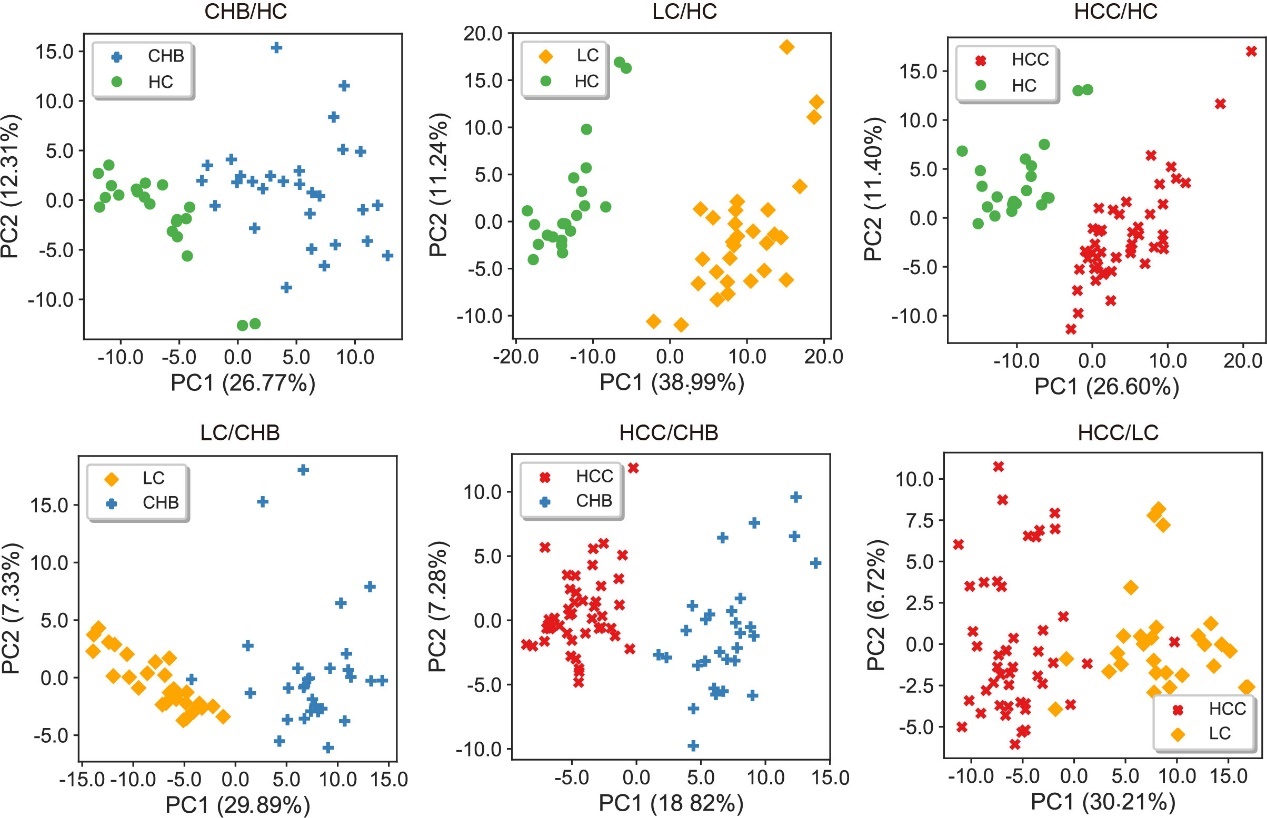


### Figure S9. Principal component analysis using DEPs identified between any two patient groups (HC, CHB, LC, HCC).


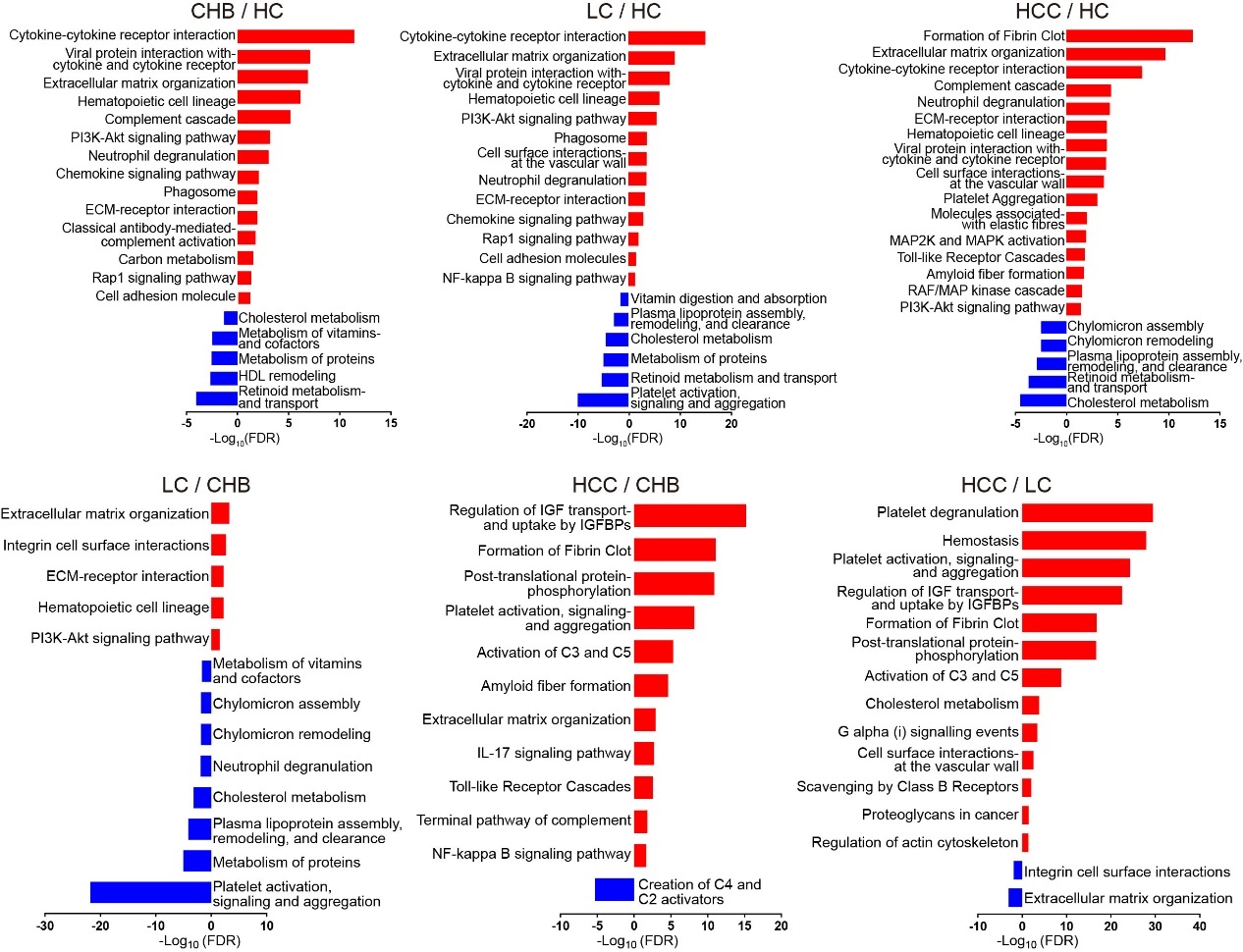


### Figure S10. Signaling pathway analysis of DEPs between any two groups of samples (HC, CHB, LC, HCC) by the STRING database (p < 0.05). The red and blue bars represent pathways that are significantly enriched in up-regulated and down-regulated proteins, respectively.


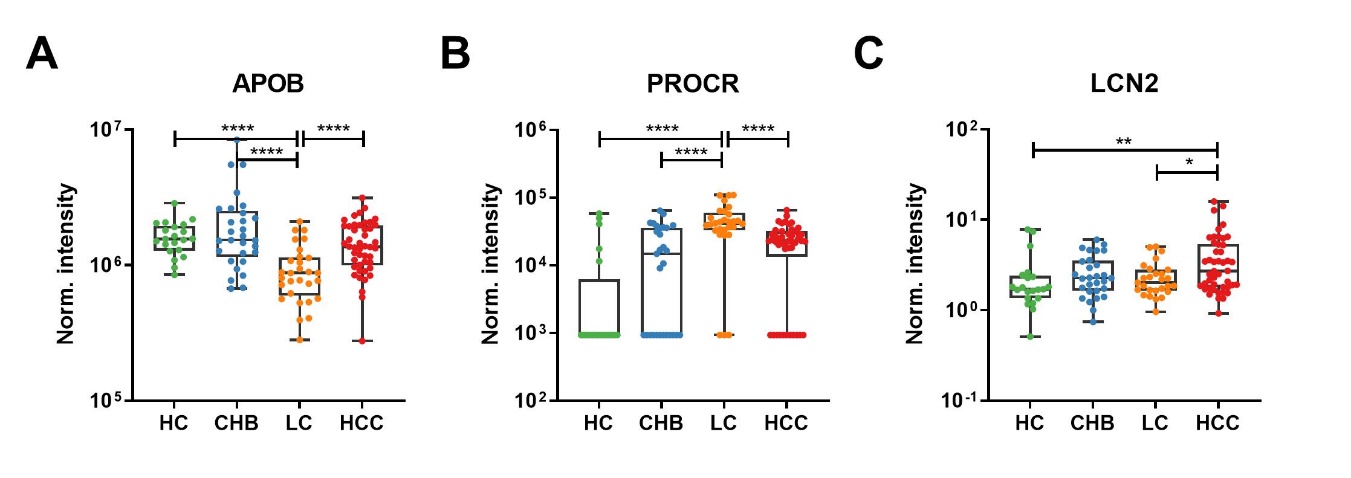


### Figure S11. The expression of representative proteins in cluster I (A), cluster II (B) and cluster III (C) in HC, CHB, LC, and HCC groups. The line and box represent median and upper and lower quartiles, respectively. * p < 0.05，** p < 0.01, **** p < 0.0001.


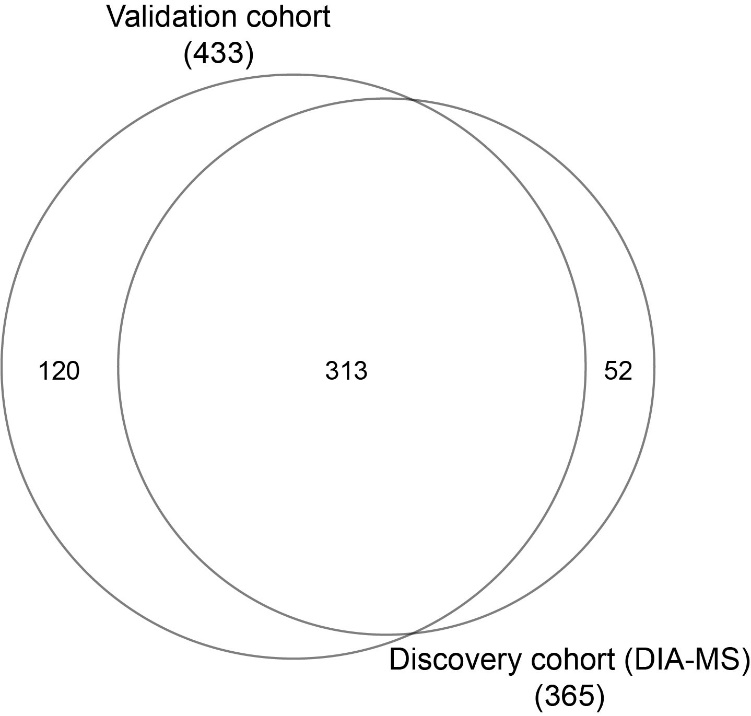


### Figure S12. Venn diagram analysis of proteins detected in the discovery and validation cohorts using DIA-MS. The Venn diagram was created using http://bioinformatics.psb.ugent.be/webtools/Venn/.


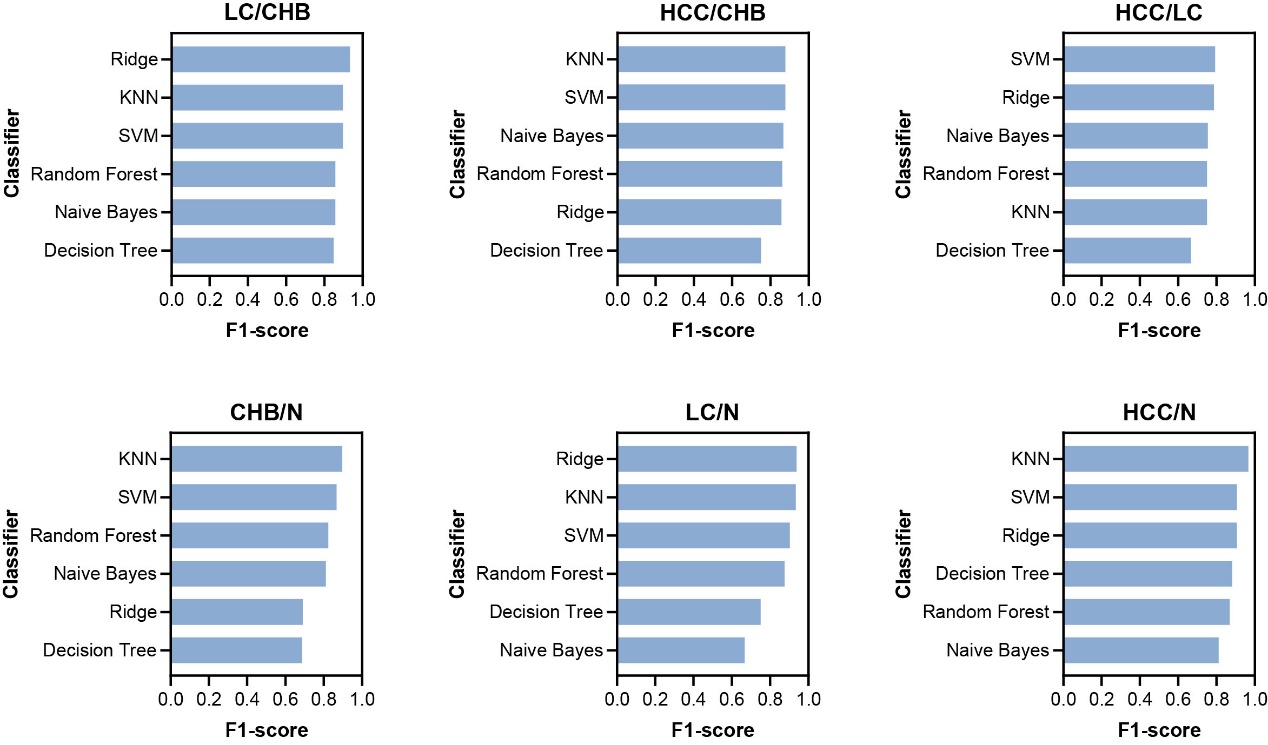


### Figure S13. Comparison of the F1-score in six advanced machine learning classifiers (Support vector machine, Ridge, KNN, Naïve Bayers, Decision Tree and Random Forest).


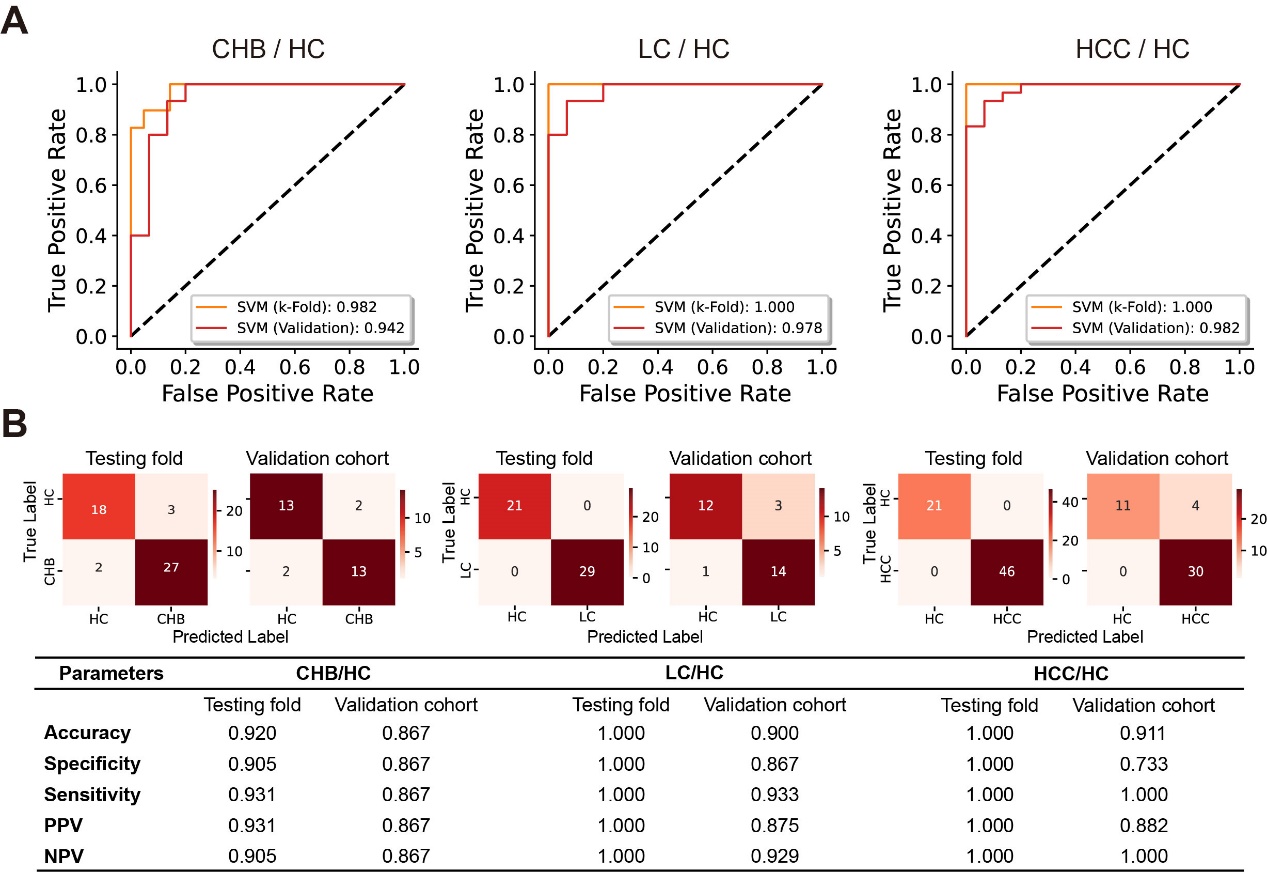


### Figure S14. The ROC curve (A) and confusion matrix performance (B) of biomarker panels in CHB versus HC, LC versus HC, and HCC versus HC across the discovery and validation cohorts.


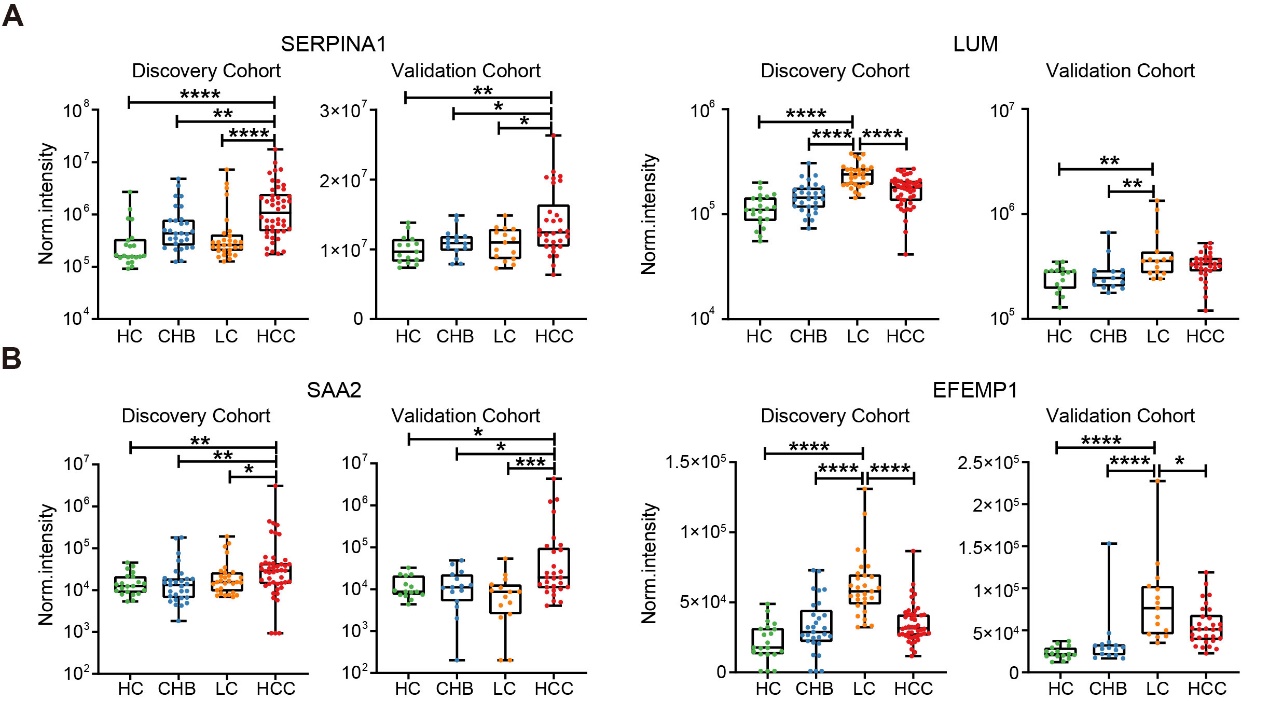


### Figure S15. Box plot analysis of protein biomarkers of HCC and LC that have been previously reported (A) and newly identified in this study (B). The line and box represent median and upper and lower quartiles, respectively. * p < 0.05，** p < 0.01, *** p < 0.001, **** p < 0.0001.


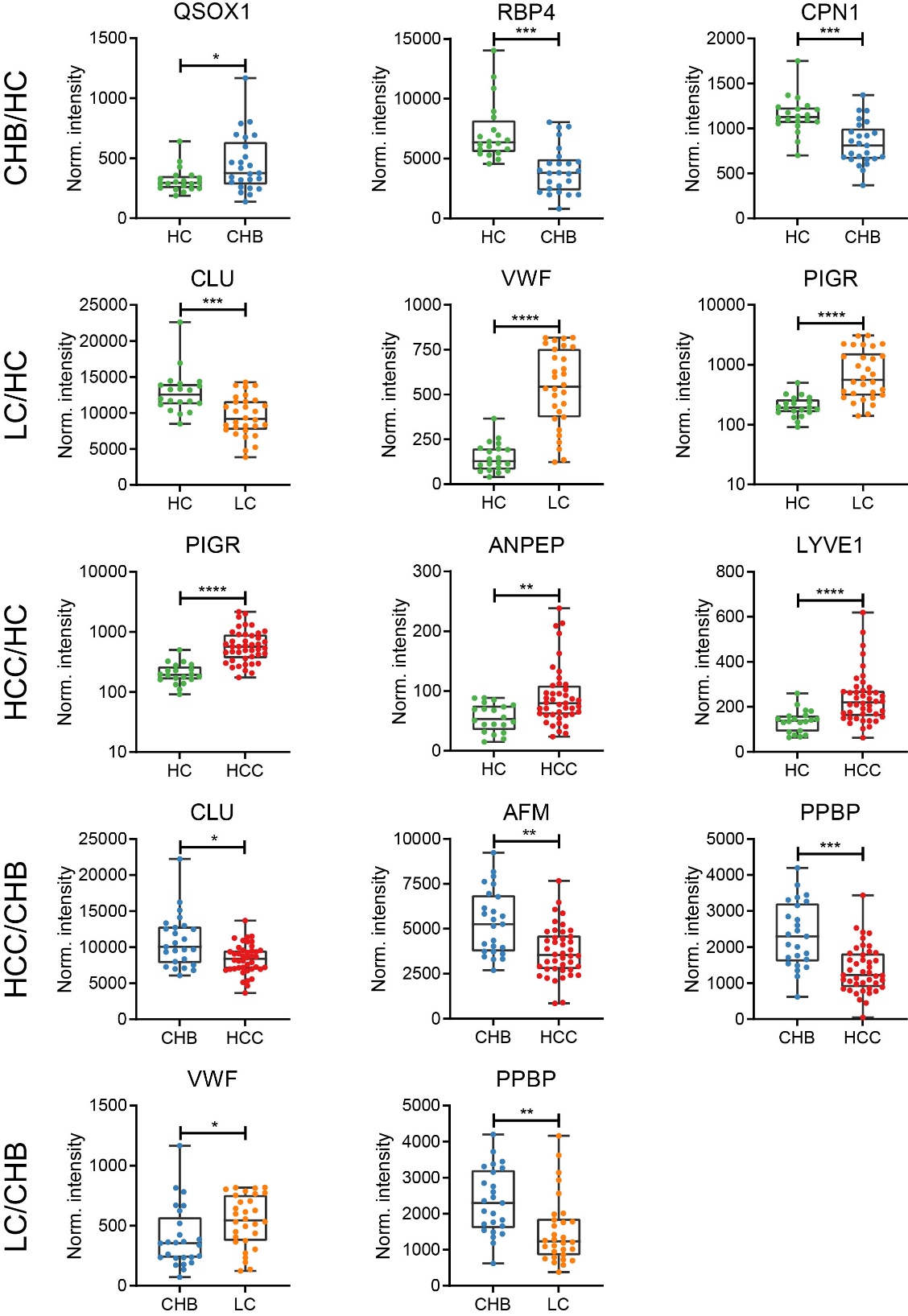


### Figure S16. Box plot analysis of representative biomarkers of CHB/HC, LC/HC, HCC/HC, LC/CHB and HCC/CHB in the additional cohort based on PRM data. The line and box represent median and upper and lower quartiles, respectively. * p < 0.05，** p < 0.01, *** p < 0.001, **** p < 0.0001.

## Supplemental Tables

| **Table S1** | List of 532 proteins detected by the antibody microarray. |
| --- | --- |
| **Table S2** | Serum sample data obtained by mass spectrometry. |
| **Table S3** | Instrument parameters of the Q Exactive HF Hybrid Quadrupole Orbitrap for data-dependent acquisition. |
| **Table S4** | Instrument parameters of the Q Exactive HF Hybrid Quadrupole Orbitrap for data-independent acquisition. |
| **Table S5** | Instrument parameters of the Orbitrap Fusion mass spectrometer for parallel reaction monitoring. |
| **Table S6** | Results of DIA-MS of 125 serum samples in the discovery cohort with detailed protein information. |
| **Table S7** | Results of DIA-MS of 75 serum samples in the validation cohort with detailed protein information. |
| **Table S8** | List of unique peptides targeted for the PRM. |
| **Table S9** | Results of PRM of 120 serum samples in the additional cohort with detailed protein information. |
| **Table S10** | List of all proteins detected in the discovery cohort. |
| **Table S11** | List of 408 biomarkers and 377 drug targets that were detected in this study. |
| **Table S12** | List of diseases enriched in the biomarkers and drug targets detected in this study. |
| **Table S13** | List of DEPs identified by antibody microarrays and DIA-MS. |
| **Table S14** | Hierarchical cluster analysis of DEPs in the discovery cohort. |
| **Table S15** | List of the 91 drug targets related to liver diseases. |
| **Table S16** | Summary of the multi-marker panels identified with machine learning. |
| **Table S17** | Performance of the multi-marker panels in differentiating liver diseases from HCs and each other in two patient cohorts. |
| **Table S18** | Protein biomarkers of HCC or LC in the multi-marker panels that have been previously reported. |
| **Table S19** | The selected biomarkers validated in the additional cohort using PRM. |
